# Blood Vessel Topography of the Feet in Selected Species of Birds of Prey and Owls

**DOI:** 10.3390/vetsci11020088

**Published:** 2024-02-14

**Authors:** Rebekka Schwehn, Elisabeth Engelke, Christian Seiler, Dominik Fischer, Hermann Seifert, Christiane Pfarrer, Michael Fehr, Marko Legler

**Affiliations:** 1 Department of Small Mammal, Reptile and Avian Medicine and Surgery, University of Veterinary Medicine Hannover, Foundation, Bünteweg 9, 30559 Hannover, Germany; michael.fehr.ir@tiho-hannover.de (M.F.); marko.legler@tiho-hannover.de (M.L.); 2 Institute for Anatomy, University of Veterinary Medicine Hannover, Foundation, Bischofsholer Damm 15, 30173 Hannover, Germany; elisabeth.engelke@tiho-hannover.de (E.E.); christiane.pfarrer@tiho-hannover.de (C.P.); 3 Institute for General Radiology and Medical Physics, University of Veterinary Medicine Hannover, Foundation, Bischofsholer Damm 15, 30173 Hannover, Germany; 4 Clinic for Birds, Reptiles, Amphibians and Fish, Justus Liebig University Giessen, Frankfurter Str. 114, 35392 Giessen, Germany; fischer@zoo-wuppertal.de; 5 Zoo Wuppertal, Hubertusallee 30, 42117 Wuppertal, Germany

**Keywords:** avian anatomy, vasculature, metatarsal, digital, contrast micro-computed tomography scan, arteriography, venography, pelvic limb, bumblefoot, raptors

## Abstract

**Simple Summary:**

Birds of prey and owls are regularly admitted in veterinary practices due to diseases of and injuries to their feet that require surgical intervention. During surgical care, it is essential to spare the blood vessels in order not to negatively influence or even interrupt the blood supply. Therefore, the topography of pedal blood vessels was compared in detail between different species of birds of prey and owls: photographs of dissected specimens and micro-computed tomography 3D reconstructions and drawings of the course of the vessels were made. Some differences in the topography of the blood vessels were revealed between the examined species. The differences in the vasculature of the foot may influence the development of circulatory-related diseases, such as pododermatitis (bumblefoot).

**Abstract:**

Birds of prey and owls are susceptible to diseases of and traumatic injuries to their feet, which regularly require surgical intervention. A precise knowledge of the blood vessel topography is essential for a targeted therapy. Therefore, the metatarsal and digital vasculature was examined in eight species of birds of prey and owls. The study included contrast micro-computed tomography scans and anatomical dissections after intravascular injection of colored latex. In all examined species, the dorsal metatarsal arteries provided the main supply to the foot and their branching pattern and number differed between species. They continued distally as digital arteries. All examined species showed a basic pattern of four collaterally located digital blood vessels per toe: a prominent artery and small vein on one side and a small artery and prominent vein on the other side. Digital veins united to form common digital veins, most of which joined into a superficial, medially located metatarsal vein. This vein provided the main drainage of the foot. The detailed visualization of the topography of pedal blood vessels will help veterinary surgeons during surgical procedures. In addition, differences in the plantar arterial arch between hawks and falcons were discussed regarding their possible influence on the prevalence of pododermatitis (bumblefoot).

## 1. Introduction

The feet are one of the most specialized parts of the body in birds of prey and owls, as they show morphological adaptations to species-specific hunting behavior and prey capture [1,2]. Therefore, diseases of and traumatic injuries to the feet seriously disable these birds. Unfortunately, disorders of the feet are common clinical problems in captive and free-living birds of prey [3]. These include tarsometatarsal and phalangeal fractures and dislocations, tendon damages and skin injuries such as abrasions, bite wounds from other animals, ring constriction lesions, and frostbite [3,4,5,6]. These injuries often require surgical intervention, including wound management [7], fracture treatment, amputation of digits [8,9], or tendon surgery [10]. Injuries to the major blood vessels may negatively affect the healing and prognosis of the diseases. Therefore, an exact knowledge of the topography of metatarsal and digital blood vessels is essential for all surgical procedures that are performed on the feet.

Pododermatitis, also known as bumblefoot, is another common disease in captive birds of prey, often requiring surgical treatment [3,11]. In contrast, the literature concerning pododermatitis in owls is rather sparse [12,13]. Husbandry conditions such as unsuitable perching surfaces [14,15], overweight [4,5], and lack of exercise [6,16,17] are discussed as possible causes for this disease. An important factor in preventing the development of bumblefoot is physical training, which significantly increases the blood circulation of the foot [17,18]. Therefore, the etiology of bumblefoot is seen in relation to circulatory disorders of the feet [6,11,17,19]. The literature shows species-specific differences in the prevalence of bumblefoot among the orders *Falconiformes*, *Accipitriformes,* and *Strigiformes*: falcons are more frequently or severely affected compared to hawks and owls [3,11]. This led us to the question whether the blood supply of the feet is related to certain anatomical features such as the blood vessel topography, which might differ between orders and, thus, may play a role in the species-specific prevalence of bumblefoot.

The general architecture of the vascular system of the feet is already known in some species of birds of prey and owls [4,20,21,22,23,24,25]. Previous studies showed that the main supply to the foot was via dorsal metatarsal arteries [4,20,21,22,23,24,25] and the main drainage of the foot was provided by a strong medial metatarsal vein [22,25]. However, interspecific variations in the branching pattern of pedal blood vessels have not been compared in detail in birds of prey and owls before. Therefore, the purpose of the study was to visualize and describe the topography of metatarsal and digital blood vessels of the hind limb in eight species of birds of prey and owls, which belong to the orders *Falconiformes*, *Accipitriformes,* and *Strigiformes* and differ in their susceptibility for bumblefoot.

## 2. Materials and Methods

Investigations were carried out on the carcasses of eight avian species: northern goshawk (*Accipiter gentilis*), common buzzard (*Buteo buteo*), peregrine falcon (*Falco peregrinus*), hybrid gyrfalcon × saker falcon (gyr–saker falcon; *Falco rusticolus* × *Falco cherrug*), common kestrel (*Falco tinnunculus*), Eurasian eagle–owl (*Bubo bubo*), long-eared owl (*Asio otus*), and barn owl (*Tyto alba*). Table 1 gives the number of the examined specimens per species, sex, and investigation method.

The hybrid gyr–saker falcons were fully grown, captive-bred animals that had been used as a control group in a former study and had been euthanized as part of this research project. The animal experiment and all necessary measures had been approved by the Animal Protection Commission of the Regierungspräsidium Giessen, Germany, with the approval number GI18/9 No. 69/2011. After completion of this project, the cadavers were made available for the present study.

All other specimens were cadavers from fully grown wild birds, which had been admitted as patients at the Department of Small Mammal, Reptile, and Avian Medicine and Surgery of the University of Veterinary Medicine Hannover, Germany. The birds had been euthanized for animal welfare reasons because they were so severely injured that they could not have been released back into the wild [26,27]. All related procedures were carried out in accordance with the German animal welfare law (Tierschutzgesetz §4, §7, §7a) as well as the Directive of the European Parliament and of the Council for the Protection of Animals Used for Experimental and other Scientific Purposes (2010/63/EU). Accordingly (Tierschutzgesetz §7), no explicit permission to conduct this study was necessary because no medical procedures or experiments were carried out while the animals were alive. This procedure was approved by the University‘s Animal Welfare Officer, confirmation TVO-2017-V-61.

All cadavers were stored at −20 °C until examination. For macroscopic dissections, they were thawed and the skin of the medial side of the femur was incised to have access to the major blood vessels of the hind limb. For the demonstration of the blood vessels, colored latex (60%, Wurfbain Nordmann GmbH, Hamburg, Germany; color: Alpina Voll-und Abtönfarbe, Alpina Farben, Ober-Ramstadt, Germany) was used. Red color was taken to demonstrate the arteries, blue for the veins. Depending on the size of the bird, 0.4 to 3 mL colored latex was injected under manual pressure either for the arteries into the ischiadic artery or for the veins into the external iliac vein. The described access to the veins had the consequence that their filling was retrograde and, compared to the arteries, an increased manual pressure had to be applied to fill the veins successfully. In case of insufficient filling of the pedal veins via the external iliac vein, the superficial medial plantar metatarsal vein was used for a second attempt except for the small species (i.e., common kestrel, long-eared owl, and barn owl). Specimens were kept in plastic bags to prevent humidity loss and stored at 6–8 °C for seven days to allow the latex to harden. Subsequently, skin, muscles, and tendons distal to the intertarsal joint were removed to expose the metatarsal and digital blood vessels. The specimens were photographed for documentation purposes (Sony SLT-A58 digital camera and Sony SAL 18–55 mm F3.5–5.6 lens, Sony Corporation, Minato, Tokio, Japan).

In addition, contrast micro-computed tomography (µCT) scans were taken. Therefore, barium sulphate (Barilux^®^ suspension, Sanochemia Diagnostics Deutschland GmbH, Neuss, Germany) in an amount of 2 to 20 mL depending on the size of the bird was injected under manual pressure into the ischiadic artery; success was verified radiographically. In this way, both the arteries and the veins were filled with contrast medium. Increased and prolonged manual pressure was necessary to successfully fill all the blood vessels. In most cases, this led to the leakage of contrast medium into the muscles of femur and tibiotarsus. µCT scans of the distal end of the tarsometatarsus and of the toes were performed using an XtremeCT (Scanco Medical AG, Brüttisellen, Switzerland) with a fixed tube voltage of 60 kV. The resolution was set to 41 µm, the integration time to 700 ms. The resulting data were evaluated, and 3D reconstruction was created using the Thermo Scientific™ Amira™ 3D visualization and analysis software (version 6.4.0, Thermo Fisher Scientific Inc., Waltham, MA, USA).

## 3. Results

The extent to which the blood vessels were filled with latex or contrast medium was dependent on the method of investigation and avian species. Therefore, the number of blood vessels visible in the specimens varied. The total number of feet examined as well as the number of blood vessels visible in the metatarsal and digital areas, respectively, are given in Table 2, Table 3 and Table 4. For a better understanding, we use the following abbreviations for arteries and veins.
TAcranial tibial arteryco_dMAcommon dorsal metatarsal arteryla_dMAlateral dorsal metatarsal arterymi_dMAmiddle dorsal metatarsal arteryme_dMAmedial dorsal metatarsal arteryla_pMAlateral plantar metatarsal arteryme_pMAmedial plantar metatarsal arteryla/me_DA1-4lateral/medial digital artery of the first–fourth toepAarterial pulvinar branchTVcaudal tibial veinme_pMVmedial plantar metatarsal veinla_pMVlateral plantar metatarsal veinme_dMVmedial dorsal metatarsal veinla_dMVlateral dorsal metatarsal veinla_dCDVlateral dorsal common digital veinme_dCDVmedial dorsal common digital veinla_pCDVlateral plantar common digital veinme_pCDVmedial plantar common digital veinla/me_DV1-4lateral/medial digital vein of the first–fourth toepVvenous pulvinar branch

### 3.1. Metatarsal Arteries

**Figure 1:**
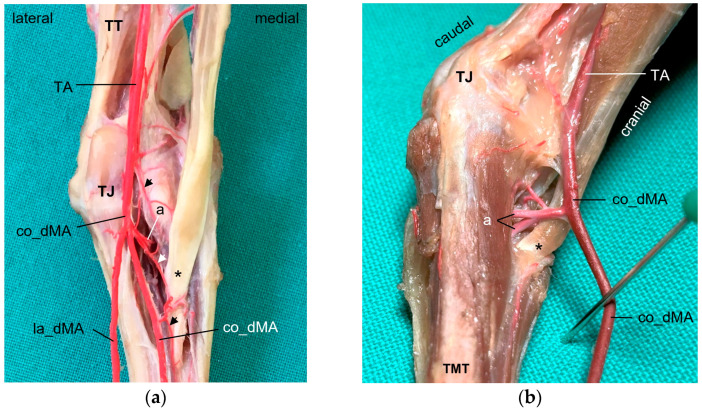
Right leg, region of intertarsal joint after removal of the skin and partial removal of muscles and tendons, arteries filled with red-colored latex. (**a**) Eurasian eagle–owl, dorsal aspect; (**b**) gyr–saker falcon, dorsolateral aspect, co_dMA displaced dorsally by a pin to show arteries to the proximal vascular foramina. TA—cranial tibial artery; co_dMA—common dorsal metatarsal artery; la_dMA—lateral dorsal metatarsal artery; a—proximal intermetatarsal arteries; TT—tibiotarsus; TJ—intertarsal joint; TMT—tarsometatarsus; *—tendon of insertion of the cranial tibial muscle; arrows—collateral arterial branches.

In all examined avian species, the cranial tibial artery (TA) provided the main blood supply of the foot. It passed the intertarsal joint dorsally lateral to the insertion of the cranial tibial muscle (Figure 1) and continued distally as a strong common dorsal metatarsal artery (co_dMA).

At the **dorsal aspect of the tarsometatarsus,** the co_dMA was located underneath the extensor tendons. Continuing distally, it divided in a species-specific manner into three dorsal metatarsal arteries. The species were divided into three groups based on this splitting.

**Figure 2:**
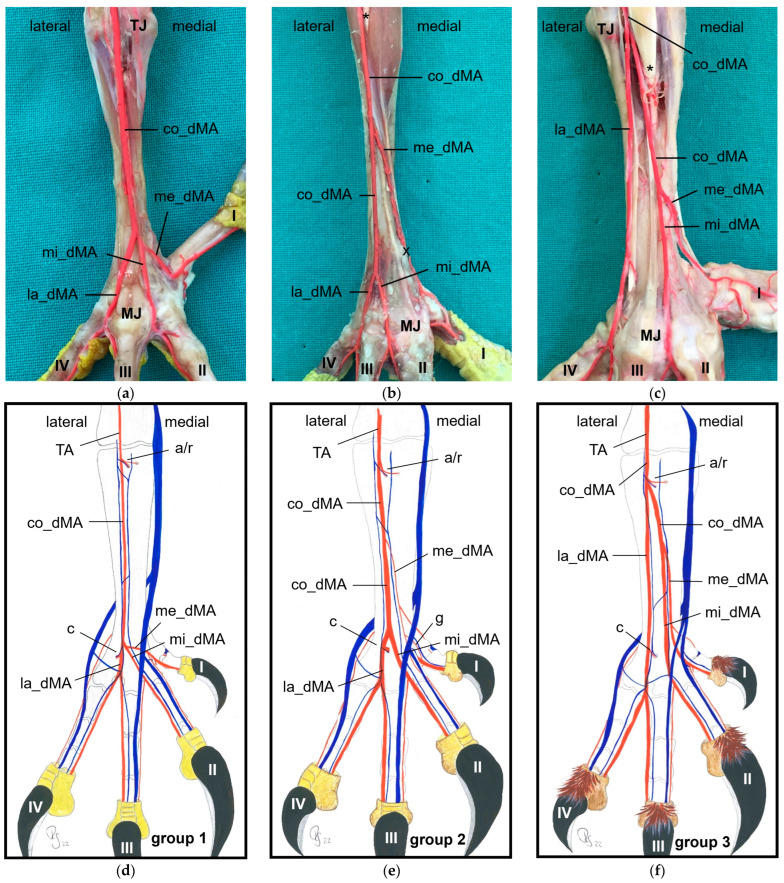
Right leg, region of tarsometatarsus, dorsal aspect. (**a**–**c**) Photographs after removal of skin and tendons, arteries filled with red-colored latex. (**d**–**f**) Semi-schematic drawings, arteries painted in red, veins in blue. (**a**,**d**) Peregrine falcon; (**b**,**e**) northern goshawk; (**c**,**f**) Eurasian eagle–owl. TA—cranial tibial artery; co_dMA—common dorsal metatarsal artery; la_dMA—lateral dorsal metatarsal artery; me_dMA—medial dorsal metatarsal artery (X: incomplete filling in (**b**)); mi_dMA—middle dorsal metatarsal artery; a—proximal intermetatarsal arteries; c—distal intermetatarsal artery; g—branch from me_dMA joining me_DA1; r—lateral proximal intermetatarsal vein; TJ—intertarsal joint; MJ—level of metatarsophalangeal joints; *—tendon of insertion of the cranial tibial muscle (removed in (**a**)); I–IV—1st–4th toe.

**Group 1** (Figure 2a,d) included peregrine falcon, gyr–saker falcon, and common kestrel. In this group, the co_dMA ran distally almost the whole distance to the metatarsophalangeal joints. It split into a lateral (la_dMA), middle (mi_dMA), and medial (me_dMA) dorsal metatarsal artery just proximal to these joints. Of these three arteries, the lateral artery was the strongest. The medial and middle artery were about the same thickness and had a very short common trunk, which was 0.5 to 1 mm long in common kestrels and mostly about 2 mm in gyr–saker and peregrine falcons.

**Group 2** (Figure 2b,e) comprised northern goshawk, common buzzard, and barn owl. In this group, the co_dMA continued distally as the major artery. A slender me_dMA branched off medially in the proximal half of the tarsometatarsus, resulting in two metatarsal arteries—one major and one minor—in the distal half. In the majority of cases, the branching point was located approximately halfway along the length of the tarsometatarsus. But in two feet of two northern goshawks and one foot of a barn owl, this artery already arose in the proximal third of the tarsometatarsus. The continuing strong co_dMA split into two arteries of about the same thickness a little proximal to the metatarsophalangeal joints, the mi_dMA and la_dMA. Thus, all three dorsal metatarsal arteries, the lateral, middle, and medial ones, were present only distally at the tarsometatarsus.

**Group 3** (Figure 2c,f) consisted of Eurasian eagle–owl and long-eared owl. In this group, the co_dMA split into two equally thick arteries directly distally to the intertarsal joint. While the lateral one ran distally in a straight course, the medial one gave rise to a third metatarsal artery in a medial direction approximately about halfway up the tarsometatarsus. In one foot of a long-eared owl, this artery already branched off in the proximal third of the tarsometatarsus. Thus, in the Eurasian eagle–owl and the long-eared owl, three parallel dorsal metatarsal arteries were present at least along the distal half of the tarsometatarsus, i.e., the la_dMA, mi_dMA, and me_dMA. In the long-eared owl, all three arteries were equally thick, while in the Eurasian eagle–owl, the medial one was slightly thinner than the other two.

For the supply of the **plantar aspect of the tarsometatarsus**, two small arteries arose from the co_dMA directly distally to the intertarsal joint (Figure 1 and Figure 2d–f), with a short common trunk in 56% of the cases. These two arteries, the medial and lateral proximal intermetatarsal arteries, passed to the plantar side through the medial and lateral proximal vascular foramina of the tarsometatarsus, where each of them divided into an ascending and a descending branch.

**Figure 3:**
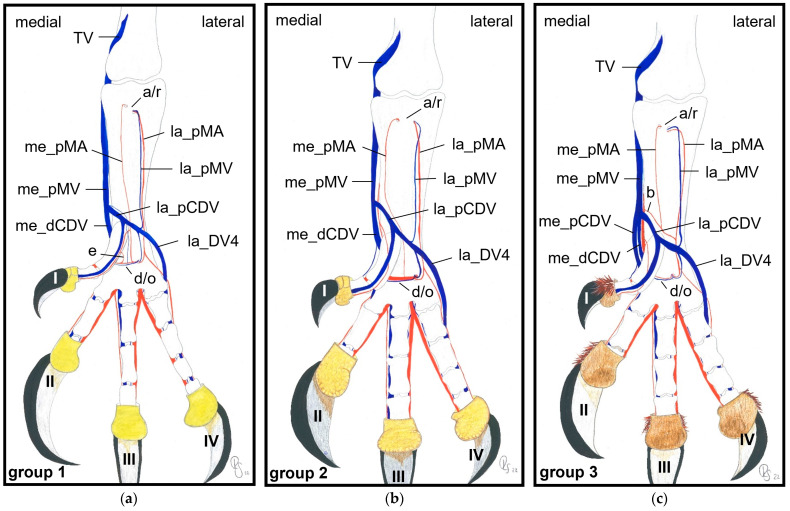
Right leg, region of tarsometatarsus, plantar aspect, semi-schematic drawings, arteries painted in red, veins in blue: (**a**) peregrine falcon, (**b**) common buzzard, (**c**) Eurasian eagle–owl. TV—caudal tibial vein; me_pMA—medial plantar metatarsal artery; la_pMA—lateral plantar metatarsal artery; me_pMV—medial plantar metatarsal vein; la_pMV—lateral plantar metatarsal vein; la_pCDV—lateral plantar common digital vein; me_pCDV—medial plantar common digital vein; me_dCDV—medial dorsal common digital vein; la_DV4—lateral digital vein of the fourth toe; a—proximal intermetatarsal arteries; b—anastomosis between me_pMA and me_dMA; d—arterial plantar arch; e—anastomosis between la_DA1 and arterial plantar arch; o—venous plantar arch; r—lateral proximal intermetatarsal vein; I–IV—1st–4th toe.

The two ascending branches turned proximally and were not investigated further in this study. The descending branches ran distally underneath the flexor tendons (Figure 3) as a thin lateral (la_pMA) and even thinner medial plantar metatarsal artery (me_pMA). This pattern was observed in all species examined, with one exception: in none of the specimens of the common kestrel was the me_pMA found. In one foot each of a common buzzard, two Eurasian eagle–owls, and two gyr–saker falcons, the me_pMA showed an anastomosis with the me_dMA in the distal third of the tarsometatarsus (Figure 3c).

**Figure 4:**
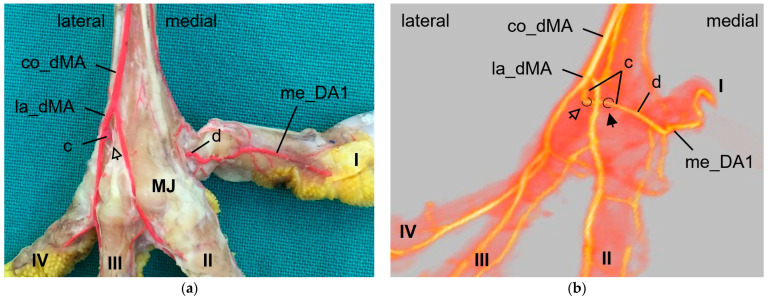
Right foot, region of metatarsophalangeal joints, dorsomedial aspect: (**a**) common buzzard, photograph after removal of skin and tendons, arteries filled with red-colored latex; (**b**) barn owl, micro-computed tomography 3D reconstruction, arteries filled with contrast medium, toes fixed with tape. co_dMA—common dorsal metatarsal artery; la_dMA—lateral dorsal metatarsal artery; me_DA1—medial digital artery of the first toe; c—distal intermetatarsal artery (runs between marked openings of the distal vascular foramen (open circles), visible in (**b**) because of transparent bone tissue in micro-computed tomography 3D reconstruction); unfilled arrow—indicates dorsal opening of distal vascular foramen; filled arrow—indicates plantar opening of distal vascular foramen; d—arterial plantar arch (visible in (**b**) because of transparent bone tissue in micro-computed tomography 3D reconstruction); MJ—level of metatarsophalangeal joints; I–IV—1st–4th toe.

Distally, at the tarsometatarsus, a second artery transversed the bone to the plantar aspect of the foot. This artery, the distal intermetatarsal artery, arose from the la_dMA (Figure 2 and Figure 4) and ran through the distal vascular foramen of the tarsometatarsus in all species studied.

After leaving this foramen on the plantar side, the artery turned medially and formed an arterial plantar arch (Figure 3 and Figure 4) underneath the flexor tendons. This arterial arch was very prominent in common buzzards, northern goshawks, and barn owls; it was less prominent in Eurasian eagle–owls, peregrine falcons, gyr–saker falcons, and common kestrels. In the long-eared owl, vessels were identified at the exit site of the distal vascular foramen on the plantar side of the tarsometatarsus, but no clear arch was visible.

The arterial plantar arch continued as the medial digital artery of the first toe (me_DA1) in the species of group 2, i.e., northern goshawk, common buzzard, and barn owl (Figure 4), which is described in greater detail below.

**Figure 5:**
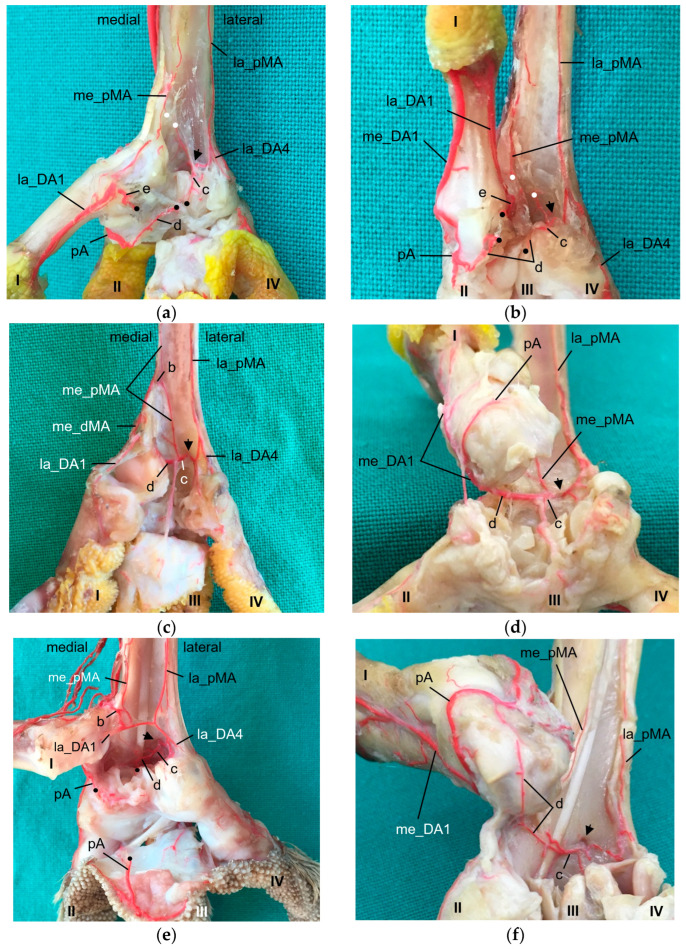
Right foot, region of metatarsal foot pad, plantar aspect after removal of skin and tendons, arteries filled with red-colored latex, first toe displaced to the left (**a**,**c**,**e**) or proximally (**b**,**d**,**f**) to show the arterial plantar arch: (**a**,**b**) peregrine falcon; (**c**,**d**) common buzzard; (**e**,**f**) Eurasian eagle–owl. me_dMA—medial dorsal metatarsal artery; la_pMA—lateral plantar metatarsal artery; me_pMA—medial plantar metatarsal artery (very small, connection marked with white dots in (**a**,**b**)); la_DA1—lateral digital artery of the first toe (incompletely filled); me_DA1—medial digital artery of the first toe; la_DA4—lateral digital artery of the fourth toe (incompletely filled); pA—arterial pulvinar branch; b—anastomosis between me_pMA and me_dMA; c—distal intermetatarsal artery passing through the distal vascular foramen (filled arrow); d—arterial plantar arch (connection to pA (**a**,**b**,**e**) and e (**a**,**b**) cut in order to dislocate the first toe proximally, cut positions marked with black dots); e—anastomosis between la_DA1 and arterial plantar arch; I–IV—1st–4th toe.

The arterial plantar arch was joined by the la_pMA (Figure 5) in all species examined. In the long-eared owl, the la_pMA joined the vessels that were visible at the exit side of the distal vascular foramen, as mentioned above. A connection of the me_pMA to the arterial plantar arch was found in some specimens of northern goshawk, common buzzard, barn owl, peregrine falcon, gyr–saker falcon, and Eurasian-eagle-owl (Figure 5a–c). In one foot of a Eurasian eagle–owl, the me_pMA joined the la_pMA (Figure 5e) instead. However, in the majority of cases including all examined specimens of the long-eared owl, the distal ending of the me_pMA remained unclear. In one foot of a peregrine falcon and one foot of a Eurasian eagle–owl, the arterial plantar arch showed an additional junction with the lateral digital artery of the first toe (Figure 3a and Figure 5a,b).

### 3.2. Digital Arteries

**Figure 6:**
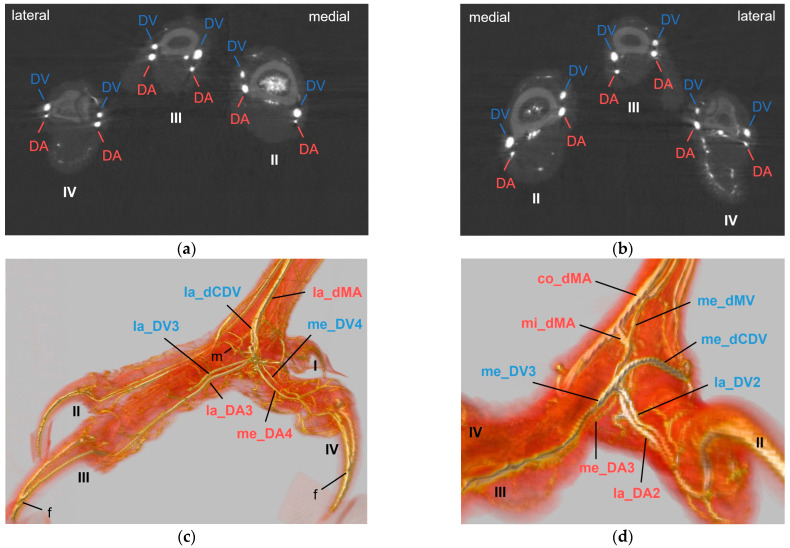
Digital arteries (labeled in red) and digital veins (labeled in blue) showing a reciprocal asymmetry in their size: (**a**,**b**) micro-computed tomography cross-section through toes II–IV, arteries and veins filled with contrast medium; (**c**,**d**) image of micro-computed tomography 3D reconstruction, arteries and veins filled with contrast medium, toes fixed with tape; (**a**) northern goshawk, right foot, proximal aspect; (**b**) northern goshawk, left foot, proximal aspect; (**c**) Eurasian eagle–owl, left foot, dorsolateral aspect; (**d**) common buzzard, right foot, dorsomedial aspect. co_dMA—common dorsal metatarsal artery; la_dMA—lateral dorsal metatarsal artery; mi_dMA—middle dorsal metatarsal artery; la_dCDV—lateral dorsal common digital vein; me_dCDV—medial dorsal common digital vein; me_dMV—medial dorsal metatarsal vein; la_DA2—strong lateral digital artery of the second toe; la_DV2—small lateral digital vein of the second toe; la_DA3—strong lateral digital artery of the third toe; me_DA3—small medial digital artery of the third toe; la_DV3—small lateral digital vein of the third toe; me_DV3—strong medial digital vein of the third toe; me_DA4—strong medial digital artery of the fourth toe; me_DV4—small medial digital vein of the fourth toe; f—arteriovenous anastomosis between digital artery und digital vein in the distal phalanx of each toe; DA—digital artery; DV—digital vein; I–IV—1st–4th toe.

The metatarsal arteries and the arterial plantar arch gave rise to the digital arteries approximately at the level of the metatarsophalangeal joints. All investigated species showed the same basic distribution pattern of the digital arteries (Figure 6): Each toe had a prominent artery on one side (i.e., medial at the first and fourth toe, lateral at the second and third toe), and a smaller artery on the other side (i.e., lateral at the first and fourth toe, medial at the second and third toe). Digital arteries continued distally along the toes directly under the skin and formed arterio-venous anastomoses with the digital veins inside the distal phalanges of the toes (Figure 6c).

The origin of the digital arteries for the supply of the first toe and the medial side of the second toe varied most between species, depending on a species-specific branching pattern of the me_dMA and on the thickness of the arterial plantar arch.

**Figure 7:**
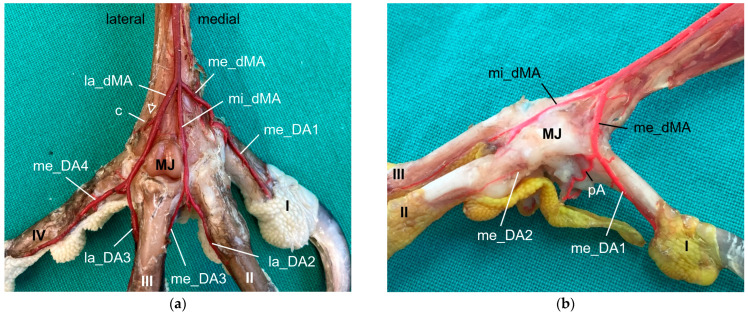
Right foot, region of the metatarsophalangeal joints after removal of skin and tendons, arteries filled with red-colored latex: (**a**) gyr–saker falcon, dorsal aspect; (**b**) peregrine falcon, medial aspect. la_dMA—lateral dorsal metatarsal artery; me_dMA—medial dorsal metatarsal artery; mi_dMA—middle dorsal metatarsal artery; me_DA1—medial digital artery of the first toe; la_DA2—lateral digital artery of the second toe; me_DA2—medial digital artery of the second toe (very small in (**b**)); la_DA3—lateral digital artery of the third toe; me_DA3—medial digital artery of the third toe; me_DA4—medial digital artery of the fourth toe; pA—arterial pulvinar branch; c—distal intermetatarsal artery passing from dorsal to plantar side through the distal vascular foramen; unfilled arrow—indicates dorsal opening of distal vascular foramen; MJ—level of metatarsophalangeal joints; I–IV—1st–4th toe.

In the examined falcons (peregrine falcon, gyr–saker falcon, and common kestrel, **group 1**), the me_dMA (Figure 2a,d) merged into the prominent medial digital artery of the first toe (me_DA1) as a direct continuation (Figure 7) and gave rise to the small medial digital artery of the second toe (me_DA2). However, the small me_DA2 (Figure 7b) was not visible in any of the examined gyr–saker falcons. The small lateral digital artery of the first toe (la_DA1) arose from the me_pMA in peregrine falcons and gyr–saker falcons (Figure 5a). It had an additional anastomosis with the arterial plantar arch only in one foot of a peregrine falcon, as mentioned before (Figure 5a,b). In the common kestrel, the la_DA1 was demonstrated only in one foot, but the origin of the artery remained unclear.

**Figure 8:**
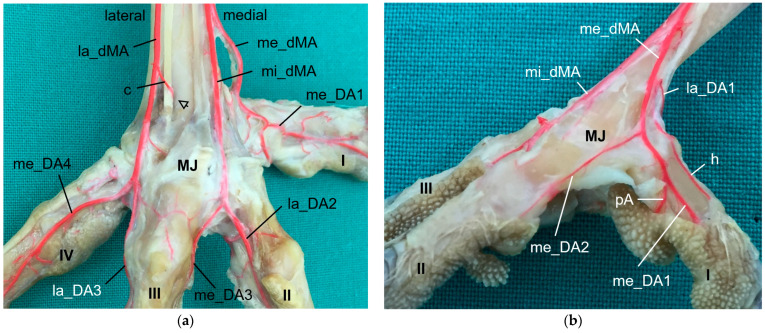
Right foot, region of the metatarsophalangeal joints after removal of skin and tendons, arteries filled with red-colored latex: (**a**) Eurasian eagle–owl, dorsal aspect; (**b**) long-eared owl, medial aspect. la_dMA—lateral dorsal metatarsal artery; me_dMA—medial dorsal metatarsal artery; mi_dMA—middle dorsal metatarsal artery; la_DA1—lateral digital artery of the first toe; me_DA1—medial digital artery of the first toe; la_DA2—lateral digital artery of the second toe; me_DA2—medial digital artery of the second toe, la_DA3—lateral digital artery of the third toe; me_DA3—medial digital artery of the third toe; me_DA4—medial digital artery of the fourth toe; pA—arterial pulvinar branch; c—distal intermetatarsal artery passing from dorsal to plantar side through the distal vascular foramen; h—duplicated me_DA1; unfilled arrow—indicates dorsal opening of distal vascular foramen; MJ—level of metatarsophalangeal joints; I–IV—1st–4th toe.

A similar distribution pattern was observed in **group 3** (Eurasian eagle-owl and long-eared owl), as the me_dMA (Figure 2c,f) divided into the strong me_DA1 (Figure 8), which was duplicated in two cases of this group, and the slender me_DA2 (Figure 8b). In addition to group 1, in most cases of group 3 in which the la_DA1 was visible, it was observed that this small vessel also arose from the me_dMA (Figure 8b), directly proximally to the respective metatarsophalangeal joint at the medial side of the tarsometatarsus. However, in one foot of a Eurasian eagle–owl the la_DA1 arose from the me_pMA instead (Figure 5e); this distribution pattern resampled that of group 1.

**Figure 9:**
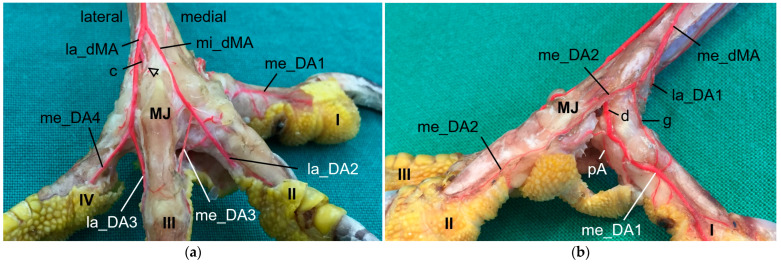
Right foot, region of the metatarsophalangeal joints after removal of skin and tendons, arteries filled with red-colored latex: (**a**) common buzzard, dorsal aspect; (**b**) common buzzard, medial aspect. la_dMA—lateral dorsal metatarsal artery; me_dMA—medial dorsal metatarsal artery; mi_dMA—middle dorsal metatarsal artery; la_DA1—lateral digital artery of the first toe; me_DA1—medial digital artery of the first toe; la_DA2—lateral digital artery of the second toe; me_DA2—medial digital artery of the second toe, la_DA3—lateral digital artery of the third toe; me_DA3—medial digital artery of the third toe; me_DA4—medial digital artery of the fourth toe; pA—arterial pulvinar branch; c—distal intermetatarsal artery passing from dorsal to plantar side through the distal vascular foramen; d—arterial plantar arch; g—branch from me_dMA joining me_DA1; unfilled arrow—indicates dorsal opening of distal vascular foramen; MJ—level of metatarsophalangeal joints; I–IV—1st–4th toe.

In **group 2** (northern goshawk, common buzzard, and barn owl), the arterial plantar arch (Figure 5c,d) was very strong, as mentioned above, and gave rise to the strong me_DA1 (Figure 5d and Figure 9). This was true for all members of this group, with a duplicated me_DA1 in six cases. In addition, the slender me_DA2 arose from the me_DA1 in the examined barn owls and in one foot of a northern goshawk and one foot of a common buzzard. In contrast, in the other feet of the examined northern goshawks and common buzzards, the me_dMA divided into three small arteries: one running to the lateral side of the first toe (Figure 5c and Figure 9b); one to the medial side of the second toe (Figure 9b); and one, as a slender anastomosis, to the strong me_DA1 (Figure 2e and Figure 9b). Unfortunately, the splitting of the me_dMA remained largely unclear in the barn owls.

Thus, only in the birds of group 2 did the me_DA1 arise from the arterial plantar arch, which was particularly strong in these species. In all other species, it originated from the me_dMA as a direct dorsal continuation. Nevertheless, a fine arterial arch also existed in the species of group 1 and 3. The me_DA1 gave rise to a strong arterial pulvinar branch to the metatarsal foot pad (pA). The pA was visible in all examined species (Figure 5d, Figure 7b, Figure 8b and Figure 9b). In two feet of two Eurasian eagle–owls, two peregrine falcons, and two gyr–saker falcons, the pA anastomosed with the arterial plantar arch (Figure 5a,b,e,f). In long-eared owls and common kestrels, this anastomosis was not present.

The arterial supply of the remaining toes was (almost) the same in all species examined. The mi_dMA (Figure 2) split into two digital arteries of different thickness; the strong one was the lateral artery of the second toe (lat_DA2) and the small one was the medial artery of the third toe (me_DA3); thus, these flanked the interdigital space of the respective toes (Figure 6d, Figure 7a, Figure 8a and Figure 9a). The la_dMA (Figure 2) divided in a similar way, but into two strong arteries (Figure 6c, Figure 7a, Figure 8a and Figure 9a): the lateral artery of the third toe (la_DA3) and the medial artery of the fourth toe (me_DA4). This splitting behavior of the mi_dMA and la_dMA was found in all species studied. The small lateral digital artery of the fourth toe (la_DA4) arose from the la_pMA in all species examined (Figure 5a–c,e), except the long-eared owl, in which the la_DA4 was not present. The origin of this artery could not be clearly determined in the examined common kestrels.

### 3.3. Digital Veins

Similar to the arteries, the veins showed a basic distribution pattern in all investigated species: Each toe had a prominent vein (i.e., lateral at the first and fourth toe, medial at the second and third toe) on one side and a smaller vein (i.e., medial at the first and fourth toe, lateral at the second and third toe) on the other side. This means that the digital veins showed a reciprocal asymmetry in their sizes in comparison to the arteries in all species studied. Figure 6 shows the different diameters of digital arteries and veins.

Generally, two digital veins united to form a common digital vein proximally to each interdigital space—a lateral and a medial vein was formed on both the dorsal and the plantar side.

**Figure 10:**
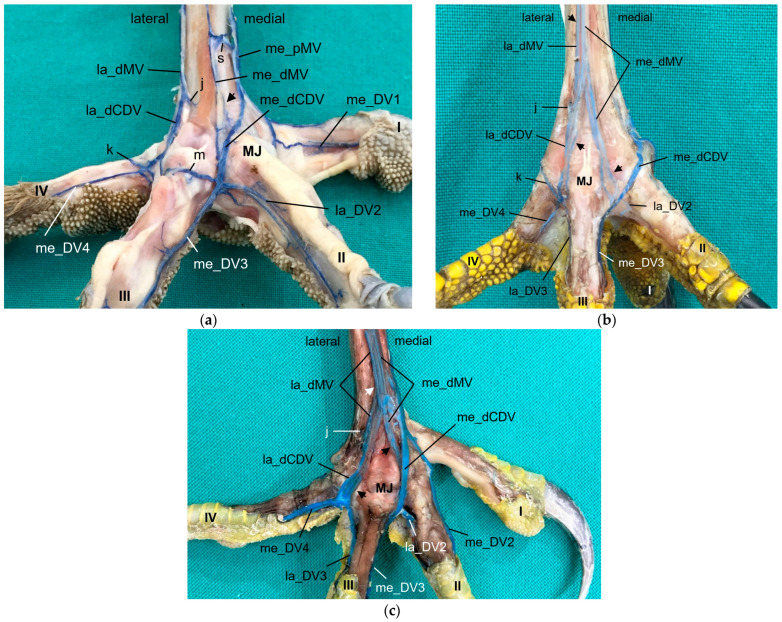
Right foot, region of the metatarsophalangeal joints, dorsal aspect after removal of skin and tendons, veins filled with blue-colored latex: (**a**) Eurasian eagle–owl; (**b**) common buzzard; (**c**) peregrine falcon. la_dMV—lateral dorsal metatarsal vein; me_dMV—medial dorsal metatarsal vein; arrows—collateral dorsal metatarsal branches; me_pMV—medial plantar metatarsal vein; la_dCDV—lateral dorsal common digital vein; me_dCDV—medial dorsal common digital vein; me_DV1—medial digital vein of the first toe; la_DV2—lateral digital vein of the second toe (incomplete filling in (**b**,**c**)); me_DV2—medial digital vein of the second toe; la_DV3—lateral digital vein of the third toe; me_DV3—medial digital vein of the third toe; me_DV4—medial digital vein of the fourth toe; j—distal intermetatarsal vein passing through the distal vascular foramen; k—anastomosis between la_dCDV and la_DV4; m—anastomosis between me_dCDV and la_dCDV; s—anastomosis between me_pMV and me_dMV; MJ—level of metatarsophalangeal joints; I–IV—1st–4th toe.

At the **dorsal aspect of the foot**, the lateral digital vein of the third toe (la_DV3) and the medial one of the fourth toe (me_DV4), both small veins, merged on the height of the metatarsophalangeal joints (Figure 6c and Figure 10). Their union created a small lateral dorsal common digital vein (la_dCDV). This vein ran proximally underneath the extensor tendons and received a small vein that entered the dorsal side of the foot through the distal vascular foramen, the distal intermetatarsal vein (Figure 10). The resulting very slender vein (Figure 10) was the lateral dorsal metatarsal vein (la_dMV), which lay deep under the extensor tendons. This pattern was true for all species examined.

The small lateral digital vein of the second toe (la_DV2) and the strong medial one of the third toe (me_DV3) joined at the level of the metatarsophalangeal joints (Figure 6d and Figure 10). Thus, they formed a strong superficial vein, the medial dorsal common digital vein (me_dCDV), which was positioned directly underneath the skin and turned to the medial side of the tarsometatarsus in all species examined (Figure 10). The small medial dorsal metatarsal vein (me_dMV) also arose from this junction (Figure 10). In contrast, the me_dMV was positioned underneath the extensor tendons.

At the level of the metatarsophalangeal joints, a dorsal veno-venous anastomosis was observed between the la_dCDV and the lateral vein of the fourth toe (la_DV4) in all species examined (Figure 10a,b) except for the common kestrel and the gyr–saker falcon. A similar anastomosis between the me_dCDV and la_dCDV was only visible in the two owl species of group 3 (Figure 10a). In one foot of a long-eared owl and one foot of a Eurasian eagle–owl, another anastomosis was present between the me_dCDV and the medial vein of the second toe (me_DV2). These anastomoses all emerged very close to the junctions of the digital veins to form the common digital veins.

**Figure 11:**
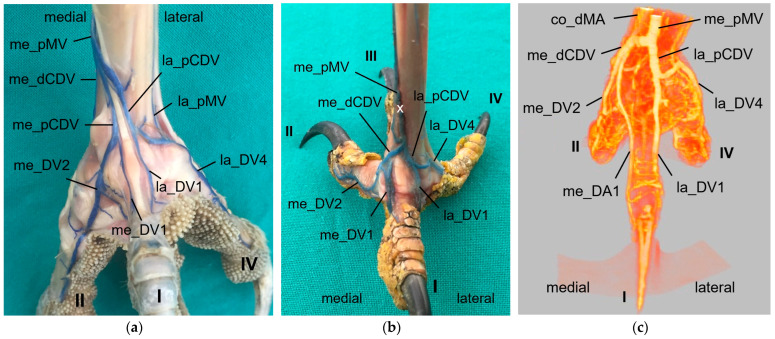
Right foot, region of the metatarsophalangeal joints, plantar aspect: (**a**,**b**) photographs after removal of the skin, veins filled with blue-colored latex. (**a**) Eurasian eagle–owl, (**b**) common buzzard, (**c**) peregrine falcon, micro-computed tomography 3D reconstruction, arteries and veins filled with contrast medium, toes fixed with tape. co_dMA—common dorsal metatarsal artery; la_pMV—lateral plantar metatarsal vein; me_pMV—medial plantar metatarsal vein (X: incomplete filling in (**b**)); me_dCDV—medial dorsal common digital vein; la_pCDV—lateral plantar common digital vein; me_pCDV—medial plantar common digital vein; la_DV1—lateral digital vein of the first toe; me_DA1—medial digital artery of the first toe; me_DV1—medial digital vein of the first toe; me_DV2—medial digital vein of the second toe; la_DV4—lateral digital vein of the fourth toe; I–IV—1st–4th toe.

**Figure 12:**
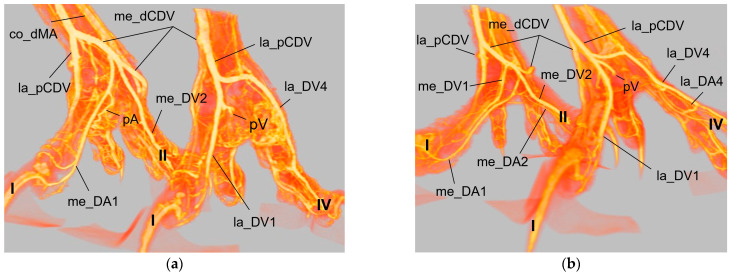
Pair of feet, region of the metatarsophalangeal joints, right plantar aspect, image of micro-computed tomography 3D reconstruction, arteries and veins filled with contrast medium, toes fixed with tape: (**a**) peregrine falcon; (**b**) northern goshawk. co_dMA—common dorsal metatarsal artery; me_dCDV—medial dorsal common digital vein; la_pCDV—lateral plantar common digital vein; la_DV1—lateral digital vein of the first toe; me_DA1—medial digital artery of the first toe; me_DV1—medial digital vein of the first toe; me_DA2—medial digital artery of the second toe; me_DV2—medial digital vein of the second toe; la_DA4: lateral digital artery of the fourth toe; la_DV4—lateral digital vein of the fourth toe; pA—arterial pulvinar branch; pV—venous pulvinar branch; I–IV—1st–4th toe.

**Figure 13:**
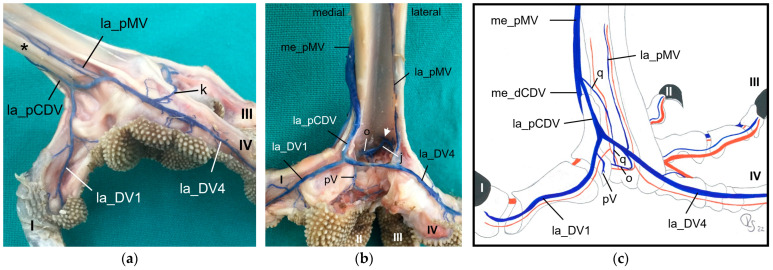
Right foot, region of the metatarsophalangeal joints and metatarsal foot pad. (**a**,**b**) Eurasian eagle–owl, veins filled with blue-colored latex: (**a**) lateral aspect, photograph after removal of the skin; (**b**) plantolateral aspect, first toe displaced to the left, photograph after removal of skin and tendons; (**c**) peregrine falcon, lateral aspect, semi-schematic drawing, arteries painted in red, veins in blue. la_pMV—lateral plantar metatarsal vein; me_pMV—medial plantar metatarsal vein; la_pCDV—lateral plantar common digital vein; me_dCDV—medial dorsal common digital vein; la_DV1—lateral digital vein of the first toe; la_DV4—lateral digital vein of the fourth toe; pV—venous pulvinar branch; j—distal intermetatarsal vein passing through the distal vascular foramen (filled arrow); k—anastomosis between la_dCDV and la_DV4; o—venous plantar arch; q—additional vein from venous plantar arch that joined me_dCDV; *—flexor tendons (removed in (**b**)); I–IV—1st–4th toe.

At the **plantar aspect of the foot**, the strong la_DV4 (Figure 11, Figure 12 and Figure 13) ran in a medial direction like an arch over the flexor tendons, connected with the strong lateral digital vein of the first toe (la_DV1) and in this way formed the strong lateral plantar common digital vein (la_pCDV). This vein stayed superficial to the flexor tendons and continued medially (Figure 11, Figure 12 and Figure 13). Subsequently, the la_pCDV joined the me_dCDV at the medial side of the tarsometatarsus in all species examined (Figure 3 and Figure 11).

A venous pulvinar branch (pV) arose either from the la_DV1 or la_DV4 or the point of their confluence (Figure 12 and Figure 13b,c). This strong branch was visible in all examined species.

**Figure 14:**
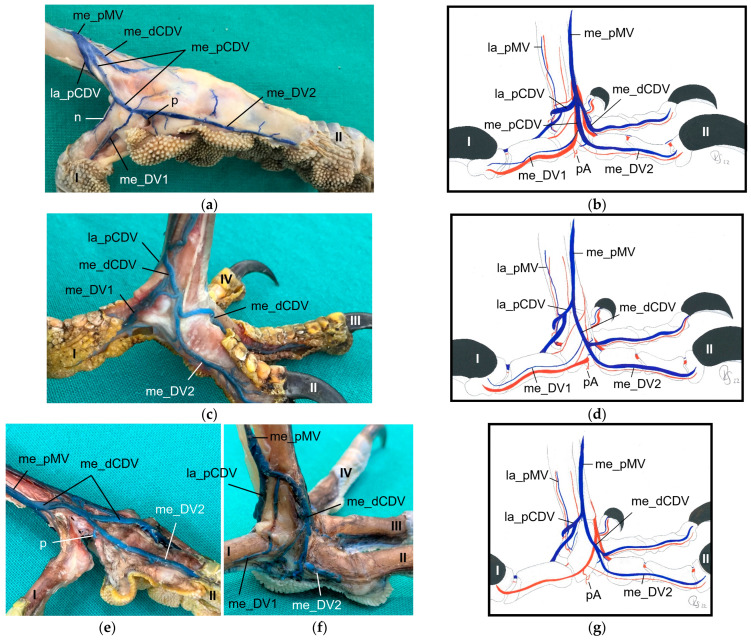
Left foot, region of the metatarsophalangeal joints, medial aspect. (**a**,**c**,**e**,**f**) Photographs after removal of skin and partial removal of tendons, veins filled with blue-colored latex; (**b**,**d**,**g**) semi-schematic drawings, arteries painted in red, veins in blue. (**a**,**b**) Eurasian eagle–owl; (**c**,**d**) common buzzard; (**e**,**g**) peregrine falcon; (**f**) gyr–saker falcon. me_pMV—medial plantar metatarsal vein; la_pMV—lateral plantar metatarsal vein; me_dCDV—medial dorsal common digital vein; me_pCDV—medial plantar common digital vein; la_pCDV—lateral plantar common digital vein; me_DV1—medial digital vein of the first toe; me_DV2—medial digital vein of the second toe; pA—arterial pulvinar branch; n—duplicated me_DV1; p—venous branch turning to plantar side forming deep venous arch; I–IV—1st–4th toe.

The small medial digital vein of the first toe (me_DV1) joined the strong me_DV2 (Figure 14a,b) to form the medial plantar common digital vein (me_pCDV). This medial vein, like the lateral one, was superficial to the flexor tendons and joined the me_dCDV at the medial side distally at the tarsometatarsus (Figure 14a,b). This pattern was seen in all feet of the owls of group 3 and of the barn owls, in which the filling of the medial digital vein of the first toe had been successful. However, in common buzzards and northern goshawks, the me_DV1 and me_DV2 joined the me_dCDV (Figure 14c,d) without the formation of a me_pCDV. The medial veins of the first and second toe opened into the common vein either in one point in most of the goshawks or with a distance of a few millimeters (up to 15 mm) in the remaining goshawks and the buzzards. In nearly all specimens of the falcon species examined, the me_DV1 could not be demonstrated; thus, only the meeting between me_DV2 and me_dCDV was observed (Figure 14e,g). In one of the examined gyr–saker falcons, the me_DV1 was visible, which opened into the common digital vein with a distance of 12 mm to the me_DV2 (Figure 14f). In three cases of group 2 and one case of group 3, the me_DV1 was duplicated (Figure 14a).

**Figure 15:**
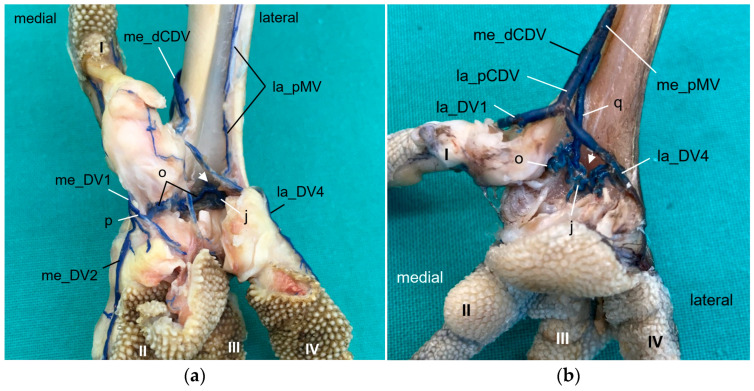
Right foot, region of the metatarsophalangeal joints and metatarsal foot pad, plantar aspect after removal of skin and tendons, veins filled with blue-colored latex. (**a**) Eurasian eagle–owl, first toe displaced proximally to show connection between venous plantar arch and me_DV1/2; (**b**) gyr–saker falcon, first toe displaced to the left. la_pMV—lateral plantar metatarsal vein; me_pMV—medial plantar metatarsal vein; la_pCDV—lateral plantar common digital vein; me_dCDV—medial dorsal common digital vein; la_DV1—lateral digital vein of the first toe; me_DV1—medial digital vein of the first toe; me_DV2—medial digital vein of the second toe; la_DV4—lateral digital vein of the fourth toe; j—distal intermetatarsal vein passing through the distal vascular foramen (filled arrow); o—venous plantar arch; p—venous branch turning to plantar side joining venous plantar arch; q—additional vein from venous plantar arch that joined me_dCDV; I–IV—1st–4th toe.

Additionally, at the plantar aspect of the tarsometatarsus, a deep venous plantar arch (Figure 3, Figure 13b,c and Figure 15) existed underneath the flexor tendons directly proximally to the metatarsophalangeal joints. This arch was formed by a small venous branch from the me_DV1 or me_DV2 or just their point of junction with the me_dCDV (Figure 14a,e and Figure 15a). It ran laterally over the plantar side deep underneath the flexor tendons and was accompanied by the arterial plantar arch (Figure 3). Directly at the tarsometatarsal bone, the distal intermetatarsal vein branched off from the deep venous plantar arch, which changed from plantar to dorsal through the distal vascular foramen of the tarsometatarsus (Figure 13b and Figure 15) and united with the la_dMV as described above (Figure 10). In common kestrels and barn owls, vessels were identified at the distal vascular foramen on the plantar side of the tarsometatarsus, but no clear venous arch was visible.

In one foot each of a peregrine falcon and four gyr–saker falcons, an additional venous vessel arose from the venous plantar arch and joined the me_dCDV in one junction with the la_pCDV (Figure 15b). It was a slender vessel in the peregrine falcon but a very strong branch in the examined gyr–saker falcons.

### 3.4. Metatarsal Veins

In the owls of group 3, as well as in the barn owls, both plantar common digital veins joined with the me_dCDV medially (Figure 3c, Figure 11a and Figure 14a,b). They formed the medial plantar metatarsal vein (me_pMV) in the distal third of the tarsometatarsus (Figure 3c, Figure 11a and Figure 14a,b) this way. In northern goshawks, common buzzards and in all examined falcon species (group 1), the union pattern was almost the same with the difference that the me_pCDV was absent (Figure 3a,b). Thus, either both corresponding digital veins or only the me_DV2 (if that one of the first toe was missing) connected individually to the me_dCDV (Figure 14c–g).

**Figure 16:**
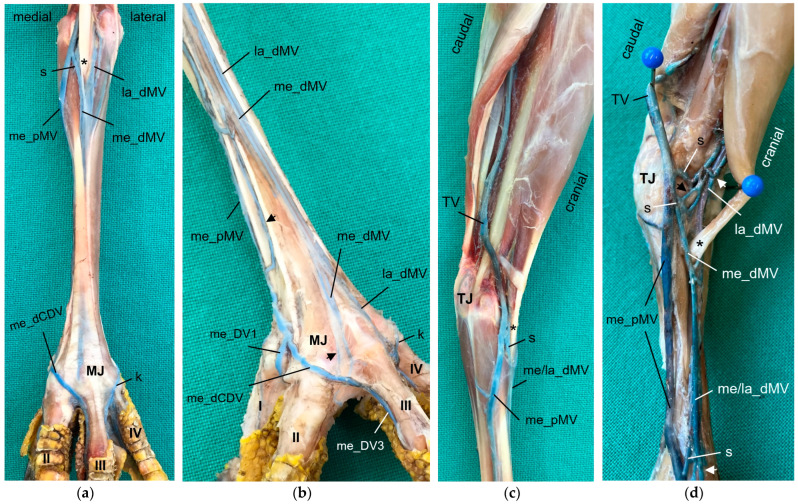
Left leg, region of tarsometatarsus, veins filled with blue-colored latex. (**a**) Common buzzard, dorsal aspect after removal of the skin; (**b**) common buzzard, dorsomedial aspect after removal of skin and tendons; (**c**) common buzzard, medial aspect after removal of the skin; (**d**) gyr–saker falcon, medial aspect after removal of skin and tendons. TV—caudal tibial vein; me_pMV—medial plantar metatarsal vein; la_dMV—lateral dorsal metatarsal vein; me_dMV—medial dorsal metatarsal vein; arrows—collateral dorsal metatarsal branches; me_dCDV—medial dorsal common digital vein; me_DV1—medial digital vein of the first toe; me_DV3—medial digital vein of the third toe; k—anastomosis between la_dCDV and la_DV4; s—anastomosis between me_pMV and me/la_dMV; TJ—intertarsal joint; MJ—level of metatarsophalangeal joints; *—tendon of insertion of the cranial tibial muscle; I–IV—1st–4th toe.

The resulting, very strong me_pMV was positioned superficially directly underneath the skin on the medial side of the tarsometatarsus and continued proximally. It changed to the dorsal side of the foot shortly before reaching the intertarsal joint and passed the joint dorsally. Proximal to the intertarsal joint, the vein turned back to the caudal side of the leg to continued proximally as the caudal tibial vein (TV), which provided the main drainage of the foot in all species examined (Figure 16).

The strong la_DV4 (Figure 3 and Figure 13) gave rise to the very slender lateral plantar metatarsal vein (la_pMV). This vein, which was located superficially at the distal end of the tarsometatarsus, passed under the flexor tendons in its proximal course (Figure 11a, Figure 13 and Figure 15a). At the proximal end of the tarsometatarsus, it transversed the lateral proximal vascular foramen as the small lateral proximal intermetatarsal vein to reach the dorsal side of the foot and join the dorsal metatarsal veins (Figure 2 and Figure 3). There were some exceptions to this course, as the la_pMV vein was not present in any of the examined common kestrels, long-eared owls, or gyr–saker falcons.

On the dorsal side of the foot at the level of the metatarsophalangeal joints, or slightly more proximal, the two dorsal common digital veins gave rise to the la_dMV and me_dMV, which were present in all species examined (Figure 10). In their proximal course, these slender dorsal metatarsal veins split into several small veins running parallel and accompanying the dorsal metatarsal arteries underneath the extensor tendons along the dorsal side of the tarsometatarsus (Figure 10 and Figure 16). The dorsal metatarsal veins showed various veno-venous anastomoses with each other as well as to the me_pMV (Figure 10a and Figure 16a,c,d).

### 3.5. Summary of the Results

**Figure 17:**
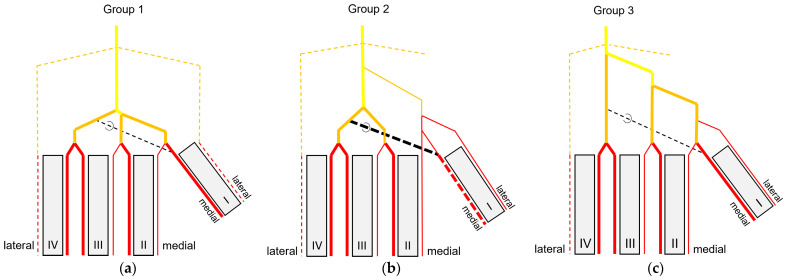
Schematic drawing of basic pattern of metatarsal and digital arteries, right foot, dorsal aspect, first toe displaced medially: (**a**) peregrine falcon, gyr–saker falcon, and common kestrel (group 1); (**b**) northern goshawk, common buzzard, and barn owl (group 2); (**c**) Eurasian eagle–owl and long-eared owl (group 3). Yellow-filled lines—common dorsal metatarsal artery (co_dMA); orange-filled lines—dorsal metatarsal arteries (la/me/mi_dMA); orange dotted lines—plantar metatarsal arteries (la/me_pMA); red-filled lines—digital arteries arising from dorsal side; red dotted lines—digital arteries arising from plantar side; black dotted lines—distal intermetatarsal artery passing through the marked (open circle) distal vascular foramen and continuing as arterial plantar arch; I–IV—1st–4th toe.

**Figure 18:**
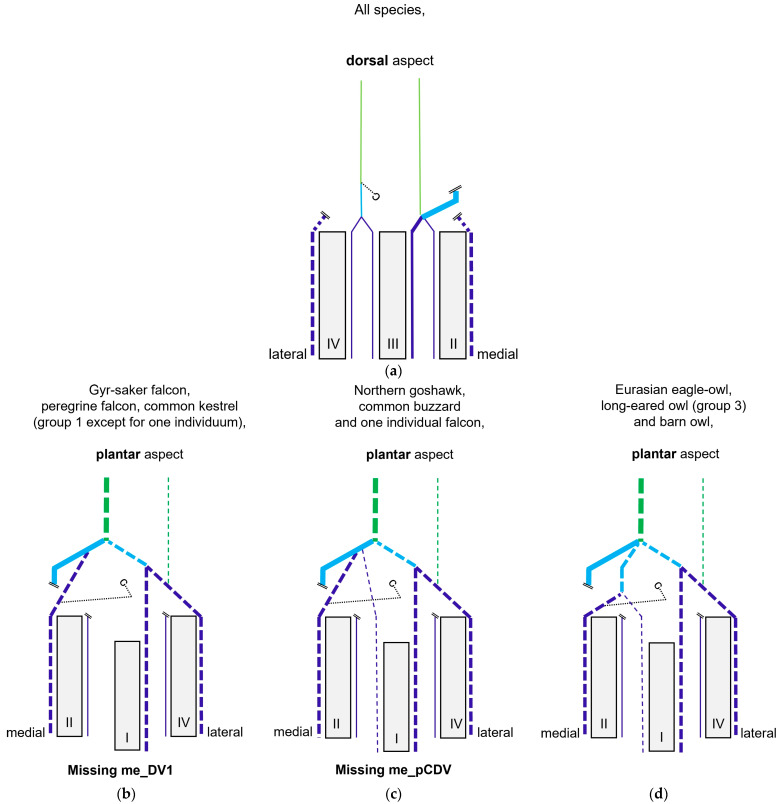
Schematic drawing of basic pattern of metatarsal and digital veins, right foot, digital veins that continue as dorsal metatarsal veins demonstrated in filled lines, digital veins that emerge to the plantar side and continue as plantar metatarsal veins in dotted lines: (**a**) dorsal aspect, all species; (**b**–**d**) plantar aspect; (**b**) gyr–saker falcon, peregrine falcon, and common kestrel (group 1 except for one individuum); (**c**) northern goshawk, common buzzard, and one individual falcon; (**d**) Eurasian eagle–owl, long-eared owl (group 3), and barn owl. Light-green-filled lines—dorsal metatarsal veins (la/me_dMV); dark-green dotted lines—plantar metatarsal veins (la/me_pMV); filled or dotted light blue lines—common digital veins (la/me_d/pCDV); filled or dotted dark-blue lines—digital veins (la/me_DV1-4); black dotted lines—distal intermetatarsal vein passing through the marked (open circle) distal vascular foramen originating from the venous plantar arch; I–IV—1st–4th toe.

In summary, the main arterial supply of the foot was provided by one common artery on the dorsal side of the tarsometatarsus in all species examined. Based on the position of the splitting of this common metatarsal artery into three dorsal metatarsal arteries, three groups of species were differentiated (Figure 17). The digital arteries arose directly from the metatarsal arteries or were prolongations of these arteries. The plantar position of the first toe required a course of the supplying arteries to the plantar side, with the origin of the digital arteries varying in a species-specific manner. In all species examined, one artery and one vein were found on the medial and lateral sides of each toe, which showed a reciprocal asymmetry of their size. In contrast to the arteries, digital veins (Figure 18) basically united to form common digital veins, which drained into four metatarsal veins. The major difference between species involved the presence of the me_DV1 and the formation of the me_pCDV (Figure 18b–d). The strong medial plantar metatarsal vein provided the main drainage of the foot. In conclusion, there was not as much difference between species in the veins of the foot as there was in the arteries.

## 4. Discussion

In order to examine the vasculature of the feet in birds of prey and owls, species were chosen for investigation because they can be taxonomically assigned to the three orders of interest: *Accipitriformes*—northern goshawk and common buzzard; *Falconiformes*—peregrine falcon, gyr–saker falcon, and common kestrel; *Strigiformes*—Eurasian eagle–owl, long-eared owl, and barn owl [28]. Out of these, the northern goshawk, peregrine falcon, and gyr–saker falcon are popular birds in falconry, while some others, e.g., the Eurasian eagle–owl, are commonly kept in zoological facilities. In addition, except for the gyr–saker falcon, all species are native to Germany and injured or ill birds are regularly admitted to clinics or wildlife rescue centers. Thus, we were able to collect at least six carcasses from each species for subsequent examination. Like several other researchers, e.g., [4,24,29], we used colored latex to fill either arteries or veins, with the advantage that latex does not leak after hardening and remains flexible, reducing the risk of breaking during dissection. In addition, we performed micro-computed tomography scans after the injection of contrast medium, allowing a 3D visualization of the blood vessel topography, even of the intraosseous courses of the vessels. The position of the arteries and veins in relation to each other became particularly clear, but in contrast to macroscopic dissection, it was more difficult to distinguish between arteries and veins due to the overlapping of the vessels. In conclusion, both methods used in this study complemented each other and were analyzed together to completely describe the topography of arteries and veins of the foot in the examined avian species.

In the distal region of the tibiotarsus and at the intertarsal joint, we found a network of collateral branches formed by the cranial tibial artery. In the literature, this arterial network is called tibiotarsal rete [22,30] or tarsal rete [29,31]. It can be derived not only from the cranial tibial artery but also from the peroneal, also named fibular, artery, and “differs species specifically in its complexity” according to Midtgård [22]. As the structures distal to the intertarsal joint were in our focus of examination, this rete was not further examined.

In all eight avian species examined, the cranial tibial artery continued distally at the tarsometatarsus as **common dorsal metatarsal artery**, which was by far the largest artery proximal at the tarsometatarsus and therefore represented the main arterial supply of the foot. This is in accordance with previous studies on some species of birds of prey, i.e., northern goshawk [4,23,25], southern caracara (*Caracara plancus*) [24], and peregrine falcon [4,23], and is consistently described in textbooks about avian anatomy for the domestic chicken (*Gallus gallus domesticus*) [30,31,32]. The present study showed that the common dorsal metatarsal artery divided into three metatarsal arteries, the **lateral, middle, and medial metatarsal arteries**, in all eight examined avian species. However, the point of division was species-specific, which allowed us to divide the species studied into three groups.

In **group 1**, which included common kestrel, peregrine falcon, and gyr–saker falcon, the common dorsal metatarsal artery split far distally just proximal to the metatarsophalangeal joints. Thus, only one dorsal metatarsal artery, i.e., the common dorsal metatarsal artery, coursed along the tarsometatarsus. A similar course and splitting of the metatarsal arteries is shown in drawings of the pedal arteries of the common kestrel by Midtgård [22] and of the peregrine falcon by Harcourt-Brown [4,23] as well as in a photograph of the southern caracara by Oliveira et al. [24]. The caracara also belongs to the family of *Falconidae* within the order *Falconiformes* [28]. This course was also described for the common kestrel in a previous study by Manno [21], who simply called the common metatarsal artery the “arteria metatarsalis dorsalis” and the resulting arteries the “common digital arteries”, due to the distal splitting and their further ramification into proper digital arteries. However, the simultaneous use of the terms “metatarsal arteries” and “common digital arteries” compared to the nomenclature in mammals [33] might suggest that there is a superficial and deep arterial system at the tarsometatarsus. However, this is not the case in birds, so we decided to use the term “metatarsal arteries” for both the common artery as well as the three arteries resulting from its splitting, which is consistent with the current avian nomenclature also used in the Nomina Anatomica Avium, e.g., [22,30,32]. In his comparative study investigating 43 different avian species, Midtgård [22] stated that the number of dorsal metatarsal arteries varied from one to three between species, and that in most bird species—including the common kestrel—just one single artery was present. However, he considered only large arteries in this assessment. Midtgård [22] labeled the three dorsal metatarsal arteries *Aa. metatarsales dorsales I*, *II,* and *III* from medial to lateral. He based this nomenclature on their original relation to the metatarsal bones before the second to fourth metatarsal bones fused into one tarsometatarsal bone during evolution [34,35]. According to Midtgård’s hypothetical pattern, it is the *A. metatarsalis dorsalis III* that remained in avian species with only one dorsal metatarsal artery [22]. However, in the domestic chicken [29,30] the terms lateral and medial dorsal metatarsal artery (*A. metatarsalis dorsalis lateralis* and *medialis*) were used for the two vessels arising from the splitting of the common dorsal metatarsal artery. The same terms were used in different species of waterfowl [36,37,38]. As this nomenclature does not require any knowledge about evolutionary theories, but reflects merely the position of the vessel, it may be easier for veterinary practitioners to handle. Therefore, we favor these terms, and we adopted the terms medial and lateral dorsal metatarsal artery and introduced the term “middle dorsal metatarsal artery”, for we had three metatarsal arteries to label. In Latin, this middle artery would be the *A. metatarsalis dorsalis media*.

Only in two species, in the domestic pigeon (*Columba livia domestica*) and the short-eared owl (*Asio flammeus*), did Midtgård [22] find three parallel arteries running distally along the tarsometatarsus. The same vessel course was seen in Eurasian eagle–owls and long-eared owls (**group 3**), which corresponds with Midtgård’s observations; these two owl species belong to the same family of *Strigidae* within the order *Strigiformes* as the short-eared owl [28]. In the little owl (*Athena noctua*), a proximal splitting in a medial and lateral metatarsal artery was reported by Manno [21]. Only further distally, he described a third artery, which split off the medial metatarsal artery, and ran to the first toe. We assume that this artery can be equated with the medial metatarsal artery of the present work.

In the representatives of **group 2**, i.e., northern goshawk, common buzzard, and barn owl, the common dorsal metatarsal artery split into two parallel arteries running along the tarsometatarsus, of which the medial one was much smaller than the lateral one. In a study on the northern goshawk, Wendt [25] described the same arterial course, but called the medial dorsal artery *A. metatarsalis plantaris*, a term which is normally used for plantar arteries beginning at the proximal vascular foramen. In contrast to this, studies on northern goshawk and peregrine falcon by Harcourt Brown [4,23] concluded that the blood vessel course of the goshawk was similar to that of the falcon, having only one dorsal metatarsal artery, the *A. metatarsalis III*. This corresponds to the description presented by Midtgård [22], who, however, only considered prominent arteries in his assessment. Although Midtgård [22] basically mentioned a second slender dorsal metatarsal artery in some species showing only one main metatarsal artery, unfortunately he did not describe in which species it occurred. Thus, the only species in which Midtgård [22] found two—equally thick—dorsal metatarsal arteries was the Humboldt penguin (*Spheniscus humboldti*). After the splitting, the lateral artery descended distally and, in our opinion, it remained the common dorsal metatarsal artery in the representatives of group 2, because it split again into two arteries at the distal end of the tarsometatarsus. This branching pattern is in accordance with that one found in the common buzzard by Manno [21], although the author used the term “common digital artery” instead of “metatarsal artery”. In the Eurasian sparrowhawk (*Accipiter nisus*), however, the division into a medial and a lateral artery was far distal, after the distal perforating branch had already left [21]. Similar to the findings in the birds of prey of group 2, the division of the common metatarsal artery into two metatarsal arteries was observed in both domestic chicken [29,30] and waterfowl [36,37,38], with the medial one being much thinner than the lateral one.

The origin of the medial and lateral **plantar metatarsal arteries** is stated to be the common dorsal metatarsal artery in most previous studies [4,29,30,31,32,36,37,38,39]. Only a few authors [22,23], who did not describe a common metatarsal artery, named the cranial tibial artery as origin. As the origin of the arteries for the plantar side was located a short distance distal to the proximal articular surface of the tarsometatarsus, we consider the common dorsal metatarsal artery to be the origin. According to veterinary anatomy textbooks [30,31], the cranial tibial artery changes its name to common dorsal metatarsal artery as soon as it is located on the tarsometatarsus. We observed a common trunk for the two proximal intermetatarsal arteries passing to the plantar side in more than half of the feet examined. In the other feet two separate arteries arose, or only one artery was visible. These different manners seemed to be individually, not necessarily dependent on the species. In the fowl, both a common trunk for two arteries [29,30,36] and the originating of two separate arteries [31,32,37,38] were described. In birds of prey, the origin of only one single artery [21,24,25], two separate arteries [4,21,23], and two arteries with a common trunk [21] were mentioned.

In order to reach the plantar side, the intermetatarsal arteries ran through the two proximal vascular foramina of the tarsometatarsus in all eight avian species examined. The term “proximal vascular foramina” was also used by the majority of authors [22,23,25,30,32,36,37,38]. Some others referred to them as “interosseal foramina” [4], or “intermetatarsal foramina” [24,29,31], as the position of the proximal foramina may correspond to the interosseous spaces between the formerly separated metatarsal bones [34,35]. Similarly, the nomenclature of the two arteries passing through the vascular foramina varied. They were labeled as “plantar tarsal arteries” [4,23,25,31,39], “plantar metatarsal arteries” [29,30], or “plantar intermetatarsal arteries” [32,36,37,38]. We consider a separate term for the arteries from their dorsal origin to their plantar proximal splitting, including the intraosseous part, would be useful for a clear description. However, both the terms “plantar tarsal arteries” and “plantar metatarsal arteries” are already used for the arteries at the plantar side of the tarsometatarsus [22,32]. “Plantar tarsal artery” is an appropriate name for a proximally running artery, because it supplies the plantar side of the tarsus. The same is true for the term “plantar metatarsal artery” for a distally running artery supplying the plantar side of the tarsometatarsus. The use of the term “plantar intermetatarsal arteries” (*Aa. intermetatarsales plantares*) for the transversing arteries, listed among others in the Nomina Anatomica Avium [32], is also confusing in our opinion, as we consider the term “plantar” inappropriate for a vessel that begins on the dorsal side of the foot. Additionally, there were not only the two proximal arteries that passed through the proximal vascular foramina, but also a distal artery that ran through the distal vascular foramen. Thus, in our opinion the terms “proximal intermetatarsal arteries” (*Aa. intermetatarsales proximales*) and “distal intermetatarsal artery” (*A. intermetatarsalis distalis*) would be appropriate as they clearly describe the course of the arteries and distinguish them from others.

Both plantar metatarsal arteries descending distally at the plantar side of the tarsometatarsus were present in seven of eight species examined; in one species, the common kestrel, only the lateral plantar metatarsal artery was demonstrated. With this observation, the question arises whether the common kestrel actually had no medial plantar metatarsal artery or whether this could not be depicted with our chosen methods due to its small size or was even due to individual variations. Manno [21] reported for the common kestrel and the little owl two plantar metatarsal arteries each, which, however, terminated approximately on half of the tarsometatarsus. In studies on the southern caracara [24], the northern goshawk [4,23,25], and the peregrine falcon [4,23], no information about the further course of the plantar arteries along the tarsometatarsus were given at all. For the domestic chicken, two arteries were described, of which only the lateral one continued distally while the medial one stayed in the region of the tarsus [29,30]. However, in two comparative references [22,32], as well as in a study about the common buzzard and the sparrowhawk [21] and in a study about the ostrich (*Struthio camelus*) [39], two metatarsal arteries were followed distally, the lateral artery being much more prominent than the medial one. Depending on the author, only the lateral [22,29,30] or both [21,31,36,37,38] descending metatarsal arteries finally joined the arterial plantar arch distally at the tarsometatarsus. We found that the lateral plantar metatarsal artery in (nearly) all specimens of all species examined connected with the plantar arch; this was true for the medial artery only for some specimens. Otherwise, the distal termination of the medial plantar metatarsal artery remained unclear in a couple of cases including all examined specimens of the long-eared owl.

The **arterial plantar arch** lay underneath the flexor tendons and was present distally at the tarsometatarsus in all species of birds of prey and owls examined, except for the long-eared owl. In the long-eared owl, we only discovered some vessels, which, however, did not form a clear arch. Here again the question arises whether the arch had not been filled due to its small size or was actually missing in the long-eared owl. When present, the arterial plantar arch originated from the artery that passed through the distal vascular foramen and that we therefore named “distal intermetatarsal artery” similar to the proximal transversing arteries, as mentioned above. The dorsal origin of the distal intermetatarsal artery was the lateral dorsal metatarsal artery in all eight species studied. This origin was also observed in some species of birds of prey and owls [4,22], in the domestic chicken [29,30], in different species of waterfowl [36,37,38], and several other bird species [22,32], even though the artery was called the third dorsal metatarsal artery in the latter studies. However, in former studies on birds of prey, i.e., northern goshawk [4,23,25] and peregrine falcon [4,23], the connection between the distal intermetatarsal artery and the arterial plantar arch was not described. In the present study, species-specific differences in the strength of the plantar arch were seen. In common buzzards, northern goshawks, and barn owls (group 2), the arch was much more prominent than in the falcon species (group 1) and in the Eurasian eagle–owl (group 3). These differences have not yet been described in species of birds of prey and owls, but have a decisive influence on the origin of the digital arteries of the first and partially of the second toe and might therefore be associated with circulatory-related pathological conditions of the feet.

In general, in the present study, we discovered two proper **digital arteries** per toe, one on the medial side and one on the lateral side. Exceptions were the gyr–saker falcon, which was missing the medial artery of toe II and the long-eared owl, which was missing the lateral artery of toe IV. Additionally, we noticed that in all toes the artery on one side (me_DA1, la_DA2, la_DA3, me_DA4) was more prominent than the artery on the other side, again in all species examined. This pattern was also shown in a comparative study in 43 species by Midtgård [22] and adopted in the Nomina Anatomica Avium by Baumel [32]. In northern goshawks and peregrine falcons, two arteries per toe were described but no differences in their size were stated by Harcourt-Brown [4]. This is in accordance with findings in the domestic duck (*Anas platyrhynchos *domesticus**) [36] and the domestic goose (*Anser anser domesticus*) [37,38]; however, only one digital artery supplying the first toe was found in these species. In the southern caracara, belonging to the family *Falconidae*, digital arteries were found medially on the first toe, and “axially” between the second and third as well as between the third and fourth toes [24]. This is similar to the distribution pattern found in the domestic chicken, in which one artery per toe was present medially at toe I and IV as well as laterally at toe II and III, and, in addition, a small opposite artery on the medial side of toe III [29,30]. This resembles the location of the main digital arteries found in the present study, plus the small medial artery of toe III. Surprisingly, in the study by Vollmerhaus and Hegner [29], an opposite artery was seen only on the third toe; on all other toes, small opposite arteries were found merely along the distal phalanges.

As mentioned above, the **medial artery of toe II and both arteries of the first toe (la_DA1, me_DA1)** showed a species-specific origin. In particular, the origin of the medial artery of the first toe depended on the size of the plantar arterial arch. In common kestrels, peregrine falcons, gyr–saker falcons, Eurasian eagle–owls, and long-eared owls (groups 1 and 3), the strong medial artery of toe I was the direct prolongation of the medial dorsal metatarsal artery, which gave also rise to the small medial artery of the second toe. Thus, in these species the medial metatarsal artery was strong while the arterial plantar arch just continued as a very small branch that only anastomosed with the medial digital artery of the first toe via the arterial pulvinar branch. The small lateral artery of toe I branched off from the medial dorsal metatarsal artery in both owl species, whereas it originated from the medial plantar metatarsal artery in peregrine falcons and gyr–saker falcons. Its origin in the common kestrel remained unclear. In this context must be noted that both methods (latex and µCT) applied in the present study reached their limits especially with blood vessels of a very small diameter in smaller avian species, i.e., common kestrel, long-eared owl, and barn owl. In some cases, vessels were not completely filled with injection mass and thus, their origin or destination could not be identified clearly. This may be the reason why in several previous studies, the smaller arteries, i.e., the lateral artery of toe I and the medial artery of toe II, were not found [21,22,24]. Although Manno [21] and Midtgård [22] used a different nomenclature, their representations depicting the large arteries showed the same origin for the me_DA1, la_DA1, and me_DA2 in falcon and owl species, as described by us. A drawing of the dorsal aspect of the pedal arteries of a peregrine falcon [4] is in total accordance with our findings in the falcon species examined. Unfortunately, the legend contradicted the drawing, which stated that the medial side of the first—and also of the fourth—toe were supplied through the distal vascular foramen. This and the authors statement that no significant differences between northern goshawks and peregrine falcons were found [4,23] did not agree with our results. Oliveira et al. [24] reported only that the common dorsal metatarsal artery gave off digital arteries, of which one ran medially along the first toe in the southern caracara. In contrast to species of groups 1 and 3, we were able to demonstrate a strong arterial plantar arch in northern goshawks, common buzzards, and barn owls (group 2), the largest part of which continued as the strong medial artery of toe I, while the medial dorsal metatarsal artery was quite small in these species. Our findings for species of group 2 were in accordance with the description for common buzzard and sparrowhawk [21], European honey buzzard (*Pernis apivorus*) [20], and northern goshawk [25]. In the study on the northern goshawk [25], a photograph suggests that the strong medial artery of toe I also was joined by the small medial dorsal metatarsal artery, which is similar to that found in the present study.

On the lateral side of the foot, a small **lateral artery of toe IV** was detected in all species examined, except for the long-eared owl. This digital artery was derived from the lateral plantar metatarsal artery. The same was also observed in the common buzzard and the sparrowhawk [21], as well as in different species of waterfowl [36,37,38], and was illustrated in a drawing showing the hypothetical original state of all metatarsal and digital arteries in birds [22]. In peregrine falcons and northern goshawks, the origin was stated to be an arterial branch from the dorsal metatarsal artery that ran through the distal vascular foramen [23], in this way, possibly describing the connection of the distal intermetatarsal artery via the plantar arch to the lateral artery of toe IV. Only in the little owl was a dorsal origin of the lateral artery of toe IV from the lateral metatarsal artery reported [21].

In all species examined, the **medial artery of toe IV and the lateral one of toe III** originated from the splitting of the lateral dorsal metatarsal artery, and the **small medial artery of toe III and large lateral one of toe II** arose from the splitting of the middle dorsal metatarsal artery. The same splitting pattern was described using old nomenclature in the European honey buzzard [20] and in a more recent study on the southern caracara [24]. A drawing of the dorsal aspect of the pedal arteries of a peregrine falcon [4] is also in accordance with our finding in the falcon species examined with respect to the splitting. The same is true for drawings of the main dorsal metatarsal arteries and their distribution pattern in the common kestrel and the short-eared owl [22]. In the northern goshawk, a photograph suggests that the pattern is similar to that; unfortunately, Wendt [25] did not explain the ramifications, but only mentioned the presence of digital arteries at the toes II to IV. Thus, the main arteries of toes IV, III, and II originated from dorsal metatarsal arteries in all birds of prey and owls examined in agreement to the available literature [4,20,22,24].

In early comparative studies on the blood supply of the avian pelvic limb, Barkow [20], Manno [21], and Hafferl [40] already defined the origin of the digital arteries from the dorsal metatarsal artery or arteries to be a **dorsal supply** of the toes. This was taken up again in a more recent study by Midtgård [22]. Although digital arteries were described as prolongations of metatarsal arteries in handbooks [30,32], in the domestic chicken, only the lateral artery of toe III (la_DA3) and the medial artery of toe IV (med_DA4) were direct continuations of the lateral dorsal metatarsal artery; the large lateral artery of toe II (la_DA2) and the large medial artery of toe I (me_DA1) had their main origin from the arterial plantar arch [22,29,30]. Only a small medial dorsal metatarsal artery also joined the lateral artery of toe II [29,30]. This pattern was defined to be a partly plantar supply [22,40]. In six different species of wild ducks, Midtgård [22] showed that the lateral artery of toe III (la_DA3) and the medial artery of toe IV (med_DA4) had their origin also from the plantar arch. According to Midtgård [22], an entirely **plantar supply** was present when all toes were provided with arteries that originated from branches passing through the proximal or distal vascular foramina of the tarsometatarsus. However, studies on the domestic goose [37,38] and domestic duck [36] observed that the lateral artery of toe II and the medial one of toe III branched off from the medial dorsal metatarsal artery. The latter one was not described in different species of the orders *Anseriformes* by Midtgård [22], as he only focused on the prominent metatarsal and digital arteries and used them for his classification into “entirely dorsal”, “entirely plantar”, and “partly plantar” supply. When all—small and prominent—metatarsal and digital arteries are taken into account, there is no entirely plantar or dorsal type of supply. Therefore, in our opinion, it would be more appropriate to speak of a mainly plantar or mainly dorsal supply. Summarizing, the present study revealed a mainly dorsal supply for common kestrel, peregrine falcon and gyr–saker falcon, Eurasian eagle–owl, and long-eared owl, and a predominantly or partly dorsal supply for northern goshawk, common buzzard, and barn owl.

Considering the results of the present study, it seems that taxonomically closely related species of birds of prey and owls show a similar course of blood vessels, as demonstrated for the course and branching of metatarsal and digital arteries in different species of the orders *Falconiformes* and *Accipitriformes* as well as the family *Strigidae*. The barn owl occupied a special position as its arterial branching pattern resembled that of the *Accipitriformes* more than that of the other owls, i.e., Eurasian eagle–owl and long-eared owl, belonging to the family of *Strigidae.* This might be explained by the fact that the barn owl belongs to another family within the order Strigiformes, the *Tytonidae*, which shows some morphological specialties in comparison to the *Strigidae* [28]. However, some even closely related species also showed specific differences in the pedal arterial supply, i.e., within the order *Charadriiformes* or *Anseriiformes* [22]. Therefore, Midtgård [22] suggested that the species-specific flexion or extension posture of the intertarsal joint in physiological movement makes either the dorsal or plantar course more convenient for the vessels, and thus different patterns have evolved in different species.

In the veins of the avian foot, a large number of venous valves occur [29,41]; unfortunately, these valves are a known problem in retrograde vein imaging, and are a possible cause of the incomplete retrograde filling of veins [22]. Reasons for this might also be the clotting of blood in the vessels or unknown previous injuries. In alignment to Vollmerhaus and Hegner [29], we observed that an increased (compared to arteries) and continuous manual pressure was necessary to overcome the venous valves and to adequately fill the veins retrogradely, especially with latex milk. Like Harcourt-Brown [4] and Vollmerhaus and Hegner [29], we found that, during the filling of the arteries, the injection material was able to pass orthogradely into veins via arteriovenous anastomoses in the distal phalanges of the toes. This effect was particularly evident when using the much less viscous X-ray contrast medium. The anastomoses in the distal phalanges were already described by Clara [42], Vollmerhaus and Hegner [29], and Schumacher [41].

Similar to the arteries, every toe had two proper **digital veins**—a lateral and a medial, one always stronger than the other one. The main digital vein lay always opposite the main digital artery in all species examined, which was seen especially well in the micro-computed tomography scans. This reciprocal asymmetry of digital arteries and veins was also observed by Midtgård [22]. In contrast to their description of arteries, Vollmerhaus and Hegner [29] found two veins per toe, with the vein not accompanied by an artery always stronger than the opposite vein. Midtgård [22] suggested that hemodynamic effects during angiogenesis could be a possible reason for the development of the reciprocal asymmetry. Similar to the development of a more dorsal or more plantar arterial supply of the toes, hemodynamic effects might cause one pathway to become prominent while the other one recedes [22].

Looking at the pictures of the vein course in the Eurasian eagle–owl, it occurred to us that each of the two digital veins that ran next to each other united into one **common digital vein**. Unlike the arteries, these common digital veins reunited in turn to form metatarsal veins. Therefore, we have introduced the term “common digital” for the veins only. This connection pattern of six out of eight proper digital veins had already been described by Vollmerhaus and Hegner [29] for the domestic chicken, but no specific term had been given to the veins. In this way, a symmetric basic pattern of digital veins was created: four common digital veins, one lateral and one medial each on the dorsal and plantar sides of the foot. This pattern was perfectly visible not only in the Eurasian eagle-owl, but in all examined owl species including the barn owl. In northern goshawks and common buzzards as well as in one single specimen of the examined falcons, a variant of this pattern was seen, as the medial plantar common digital vein was missing. The two corresponding proper digital veins joined into the medial dorsal common digital vein separately instead. This observation is in accordance with a drawing showing the pedal veins of the medial side of a foot of a northern goshawk [4], even if the nomenclature used is different to ours. In all falcons examined except one (see above), the medial digital vein of the first toe was missing. At this point we cannot tell whether the vein was just not filled or actually missing which would imply a difference in the venous branching pattern between species of the order *Accipitriformes* and *Falconiformes*. It should be noted that, also due to the variants in the branching pattern of the veins, the different species were grouped, but this did not completely coincide with the grouping based on the arterial distribution pattern. However, venous differences were only minor compared to arterial ones.

In order to join the lateral vein of toe I and to form the lateral plantar common digital vein, the strong lateral vein of toe IV curved medially superficial to the flexor tendons in all species examined. This arch-like venous course was shown in drawings made by Vollmerhaus and Hegner [29] as well as Midtgård [22]. However, this prolonged curved part of the lateral vein of toe IV was called “superficial venous plantar arch”, a term we omitted in the present study. Therefore, we would like to propose to keep the term “digital vein” for the arch-like part of the lateral vein of toe IV and to introduce the term “lateral plantar common digital vein”, after the connection with the lateral vein of toe I. We believe this would better reflect the symmetrical pattern of the venous system with four common digital veins. Until now, the superficial venous plantar arch was described to be the main plantar vein, into which the digital veins joined [4,22,32]. Vollmerhaus and Hegner [29] described the course of pedal veins in detail in the domestic chicken. They recognized the formation of common digital veins in three locations without using the term. Due to the fact that in the chicken the respective lateral vein of toe I and of toe IV joined separately into the medial superficial plantar metatarsal vein, Vollmerhaus and Hegner [29] described the curved part of the lateral vein of toe IV as superficial venous plantar arch. In drawings from Midtgård [22] of a gull and a raven, not only the la_DV4 entered into the superficial plantar arch; the med_DV3 or lat_DV1 did also.

As mentioned above, the common digital veins reunited to form metatarsal veins. In the present study, we discovered four metatarsal veins in most species examined. On the dorsal side of the tarsometatarsus, the lateral dorsal common digital vein created by the union of the la_DV3 and me_DV4 became the **lateral dorsal metatarsal vein** after the distal intermetatarsal vein was added. This branch passed through the distal vascular foramen and originated from the plantar venous arch deep under the flexor tendons in all species examined. This pattern was also observed in the domestic chicken [29]. The **medial dorsal metatarsal vein** originated from the medial dorsal common digital vein, at or only very shortly after the union of la_DV2 and me_DV3 in all avian species examined. While the origin of the medial dorsal metatarsal vein was consistent with the findings in the domestic chicken [29], its further course differed. The medial dorsal metatarsal artery joined into the lateral one in the domestic chicken creating a common dorsal metatarsal vein that continued proximally [29]. The latter was also mentioned in the northern goshawk [25]. In contrast to this description, the medial dorsal metatarsal vein ran separately proximally towards the proximal vascular foramina in all species examined in the present study. The different findings might be due to several venovenous anastomoses between the two dorsal metatarsal veins, which might suggest a confluence of the two veins. Additionally, various venovenous anastomoses to the large medial plantar metatarsal vein were observed in the species examined, which is in agreement with results in the domestic chicken [29]. These anastomoses may explain why, in two studies [25,29], the common dorsal metatarsal vein was reported to continue proximally along the tibiotarsus as cranial tibial vein. However, Baumel [32] stated that the common dorsal metatarsal vein joined the medial superficial plantar metatarsal vein in the domestic chicken. Thus, differences in the course of metatarsal veins between studies or species mentioned above might reflect actual differences but could also be associated with this frequently cross-linked metatarsal venous system leading to different interpretations by the authors. In accordance with the findings of Vollmerhaus and Hegner [29], we found a high number of venous collaterals of the dorsal metatarsal veins. The destination of the medial and lateral dorsal metatarsal veins remained unclear in the present study, because the area proximal to the tarsus was not investigated further. In the literature, a venous rete in the dorsal region of the intertarsal joint was reported, similar to the (tibio)-tarsal arterial rete. The venous rete was formed by collaterals of the dorsal veins and was also named *rete mirabile* [29,31,32].

On the plantar side of the tarsometatarsus, we discovered two metatarsal veins, the large medial plantar metatarsal vein and the small lateral plantar metatarsal vein, in almost all species studied. This observation is in line with other reports [22,29,32]. Exceptions of this pattern were seen in long-eared owls, gyr–saker falcons, and common kestrels, in which the **lateral plantar metatarsal vein** was not present. Here again, the question arises whether the vein was actually absent or whether it was just not depicted because of its small size. In all species examined in which the lateral plantar metatarsal vein was present, the origin of it was the prolonged and curved part of the lateral vein of toe IV. Previously, this part of the vein was called the superficial plantar venous arch, and described as the origin of the lateral metatarsal vein [4,22,29,32]. If present, the lateral plantar metatarsal vein continued proximally underneath the flexor tendons, passed through the lateral proximal vascular foramen as lateral proximal intermetatarsal vein and joined the common dorsal metatarsal vein on the dorsal side. The same course was described for the domestic chicken, but the vein was named the “*deep* plantar metatarsal vein” [29]. This was due to the observation that a second lateral plantar metatarsal vein was found on the lateral side of the tarsometatarsus in the domestic chicken [29]. The authors described this superficial vein as a thin cutaneous vein. According to Vollmerhaus and Hegner [29], in the domestic chicken, this vein also arose from the “superficial plantar arch”. We did not find this superficial lateral plantar metatarsal vein in any of the specimens examined. Therefore, we could omit the termini “superficial” and “deep” because there was only one plantar metatarsal vein on the lateral side. However, according to Baumel [32], Harcourt-Brown [4], and Midtgård [22], the lateral plantar metatarsal vein joined the caudal tibial vein proximal to the intertarsal joint, and a vein passing through the proximal foramen of the tarsometatarsus was not mentioned. This indicates that these authors might describe the same superficial vein as Vollmerhaus and Hegner [29].

On the medial side of the tarsometatarsus, one very large **medial plantar metatarsal vein** was present in all avian species studied. This vein was formed by the union of at least two common digital veins, i.e., the medial dorsal and the lateral plantar common digital vein. In owls, the medial plantar common digital vein was added to that union. The description and the drawings in the majority of previous publications agreed with our findings. However, they described that after the major digital veins had joined into a superficial plantar arch; this arch continued proximally as the medial superficial plantar metatarsal artery [4,22,29,32].

We also found a **venous plantar arch** deep underneath the flexor tendons, which created a connection from a medial digital vein (toe I or toe II), except for long-eared owl and common kestrel, to the distal intermetatarsal vein, which passed through the distal vascular foramen. From this foramen, the arch also connected to the lateral vein of toe IV. Only in some gyr–saker falcons and peregrine falcons did we observe a strong additional vein connecting the deep venous arch to the me_dCDV and the me_pMV. In the domestic chicken, the deep plantar arch also had a medial connection to one of the medial common veins joining into the medial superficial plantar metatarsal vein and a lateral connection to the venous branch passing through the intermetatarsal distal foramen [29]. In the domestic chicken, the deep plantar arch additionally received interdigital veins from the common veins of la_DV2 and me_DV3 and of la_DV3 and me_DV4, respectively [29]. However, the connection to the curved lateral vein of toe IV in the superficial plantar arch, which we found in the birds of prey and owls investigated in the present study, was not described in the domestic chicken. Former studies on birds of prey and owls did not mention a deep venous arch at all [4,22,25].

The medial plantar metatarsal vein provided the main drainage of the foot and continued proximally as caudal tibial vein after passing the intertarsal joint, which was in agreement with previous studies [22,25,29,30,31,32].

## 5. Conclusions

In summary, the present study described and visualized the topography of metatarsal and digital blood vessels of the avian foot in detail in eight species of birds of prey and owls. One–three large arteries on the dorsal side as well as one large vein on the medial side of the tarsometatarsus provided the main supply and drainage of the entire foot. Species-specific variations were found, especially in the branching and number of dorsal metatarsal arteries, supply type of the toes (mainly dorsal or mainly plantar), and junction pattern of common digital veins. In most species studied, similar vascular courses were often observed in taxonomically more closely related species. In contrast, the analogous branching pattern of the arteries in the barn owl and in the examined *Accipitriformes* might have evolved convergently.

Among captive birds of prey, injuries to the feet occur much more frequently in birds used for hunting in falconry than in birds kept in aviaries [4]. Just like free-living birds of prey, the former use their feet to capture prey [1,2], which are therefore particularly exposed to traumatic injuries, e.g., bite wounds caused by prey or other predators [3,4]. This results in clinical relevance, as injuries to main blood vessels could seriously impair or even interrupt the blood supply of the entire foot. Dorsal metatarsal arteries are covered only by skin and the relatively thin tendons of the digital extensors; therefore, they might be more exposed to injuries than arteries that are covered by muscles. Traumatic injuries often require surgical intervention on the feet [7,8,9,10], for which an exact knowledge of the topography of pedal vessels is essential. For example, due to the cutaneous scales, even large vessels located directly under the skin (i.e., the metatarsal veins as well as digital arteries and veins) are not easily visible to surgeons. In the present study, detailed photographs of the dissected specimens and 3D visualizations of computed tomography scans show the topography of metatarsal and digital blood vessels and may help to operate in a vessel protective way and reduce injuries to blood vessel during surgical procedures on the avian foot. This may improve the chance of complete healing of injuries and diseases of the feet after surgical treatment. Full foot function is essential not only for hunting birds in falconry, but also for rehabilitated birds, which are to be released back into the wild.

Pododermatitis (bumblefoot) is a common disease in birds of prey and owls [3,4,5]. The disease affects the area of the plantar metatarsal pad and is characterized by devitalization of the skin, followed by bacterial invasion [6,19,43]. Poor blood circulation results in a reduced ability of the skin of the feet to respond to infection [11]. Therefore, the etiology of bumblefoot is seen in relation to circulatory disorders of the feet [4,6,11,17,19]. Bumblefoot occurs much more frequently in captive than in free-living birds [3,11]. Therefore, husbandry conditions as unsuitable perching surfaces [14,15], overweight [4,5], and lack of exercise [6,16,17] are discussed as possible causes. Studies show that the incidence of bumblefoot is lower in falcons trained twice a day compared to those trained only once a day [17,44]. The blood flow to the skin of the feet is significantly increased in falcons during physical exercise, resulting in a higher temperature of the skin [17,18]. An increased number of pododermatitis cases occur especially in trained falcons after an abrupt cessation of flight activity [6,19,44,45]. This is the case, for example, at the end of the hunting season, when birds are not gradually detrained before resting for molting. The same sudden reduction in activity is experienced by a bird captured in the wild [6,19,44,45]. Thus, wild-caught falcons are more susceptible to bumblefoot than captive-bred falcons [6,19]. Heidenreich [6,19] explains this referring to highly trained athletes, who develop massive cardiovascular problems and an excess cardiac capacity when they suddenly decrease their activity level. This could be similar in birds of prey, leading to edema in the feet and secondary ischemic pressure necrosis [6,19]. Among birds of prey, falcons have the highest incidence of pododermatitis: they contract the disease more often [6,11,19,46] or more severely than hawks and respond less well to treatment [4]. Behavioral differences between species in flight activity and hunting behavior might be possible reasons for these species-specific variations in the prevalence of bumblefoot: falcons are mainly “long-distance hunters”; this could mean a greater discrepancy in physical training and cardiovascular fitness level between an active and inactive bird compared to the contrasting “short-distance hunting” accipiters and thus explain a higher susceptibility for the development of cardiovascular disorders [4,6,19]. The results of our anatomical study can be seen in connection with these findings. The examined species of the order *Accipitriformes* had a partly plantar supply and showed a strong deep plantar arch beneath the flexor tendons. The arterial plantar arch was located below the metatarsal pad. We conclude that the examined species of the order *Accipitriformes* might have an improved blood supply in the region of the metatarsal pad and therefore a lower prevalence for the development of pododermatitis. In contrast, the examined species of the order *Falconiformes* had a mainly dorsal arterial supply and showed a slender plantar arch. This might result in a worse blood supply of the region of the metatarsal pad. Additionally, there was a large plantar vein from the plantar venous arch joining the medial plantar metatarsal vein, which we only found in peregrine falcons and gyr–saker falcons. Since it was positioned on the plantar side of the foot underneath the flexor tendons, this vein might be compressed during perching, which could impair the venous drainage of the region of the metatarsal foot pad. The examined species of the order *Strigiformes* also had a mainly dorsal arterial supply. The prevalence of bumblefoot is described to be lower in owls than in falcons [3,11]. However, conclusions about the influence of vascular anatomy on the development of the disease are difficult to draw because the literature concerning owls is rather sparse [12,13]. The prevalence of bumblefoot varies in species within the order *Accipitriformes*; unlike accipiters, eagles and vultures show a higher susceptibility to pododermatitis [6,19]. As we did not examine eagles and vultures in this study, conclusions in regard to the topography of blood vessels cannot be drawn. Thus, an investigation of the pedal blood vessels in these species would be useful for future research.

We only demonstrated two strong pulvinar branches, which we assume to be the main ones: the main arterial pulvinar branch supplied the metatarsal pad arising from the medial side (me_DA1), while the main drainage was via a pulvinar vein at the lateral side (la_DV1/4) of the foot. Although a detailed study by Vollmerhaus and Hegner [29] is available on the blood supply of the metatarsal pad in the domestic chicken, a comparative description of pulvinar branches in birds of prey and owls has not yet been made. Therefore, we have designed a more detailed study to follow, on the blood supply of the metatarsal pad, including the microvascularization of the skin of the foot sole. Additional methods such as corrosion casts and histological examinations are to be used to better demonstrate small blood vessels of the skin. These further investigations are necessary to better identify a possible species-specific correlation between the blood vessel topography of the foot and the susceptibility for the development of pododermatitis.

## Figures and Tables

**Table 1:** Number of examined specimens per species, sex, and investigation method (dissection after injection (latex) and contrast micro-computed tomography (µCT) scans).

	Latex			µCT			Total
	Total	Male	Female	Total	Male	Female	
Northern goshawk	5	2	3	3	1	2	8
Common buzzard	7	4	3	3	1	2	10
Peregrine falcon	4	2	2	4	1	3	8
Gyr × saker falcon	5	5	0	3	3	0	8
Common kestrel	3	2	1	3	2	1	6
Eurasian eagle–owl	4	2	2	4	2	2	8
Long-eared owl	3	2	1	3	2	1	6
Barn owl	4	3	1	3	2	1	7

**Table 2:** Examination of metatarsal arteries and veins: number of feet examined per avian species after latex injection and dissection, and number of the respective blood vessel that was visible.

	Northern Goshawk	Common Buzzard	Peregrine Falcon	Gyr × Saker Falcon	Common Kestrel	Eurasian Eagle–Owl	Long-Eared Owl	Barn Owl
Total number of feet examined for metatarsal arteries	3	4	3	3	3	3	3	3
*Number of visible metatarsal arteries filled with latex*								
common dorsal artery (co_dMA)	3	4	3	3	3	3	3	3
lateral proximal intermetatarsal artery	3	4	3	3	3	3	2	1
medial proximal intermetatarsal artery	3	3	2	3	3	3	2	1
medial dorsal artery (me_dMA)	3	4	3	3	3	3	3	1
middle dorsal artery (mi_dMA)	3	4	3	3	3	3	3	3
lateral dorsal artery (la_dMA)	3	4	3	3	3	3	3	3
distal intermetatarsal artery	3	4	3	3	3	3	2	3
lateral plantar artery (la_pMA)	3	4	3	3	2	3	2	2
medial plantar artery (me_pMA)	2	4	3	3	0	2	1	1
Total number of feet examined for metatarsal veins	3	6	3	4	1	4	3	4
*Number of visible metatarsal veins filled with latex*								
medial plantar vein (me_pMV)	3	6	3	4	1	3	3	4
lateral plantar vein (la_pMV)	2	4	1	0	0	3	0	1
lateral proximal intermetatarsal vein	2	2	1	0	0	3	0	2
lateral dorsal vein (la_dMV)	3	6	2	4	1	2	2	3
distal intermetatarsal vein	3	6	1	2	1	3	2	3
medial dorsal vein (me_dMV)	2	5	2	3	1	2	2	3

**Table 3:** Examination of digital arteries: number of feet examined per avian species and investigation method (dissection after injection (latex) and contrast micro-computed tomography (µCT) scans), and number of the respective blood vessel that was visible.

	Northern Goshawk		Common Buzzard		Peregrine Falcon		Gyr × Saker Falcon		Common Kestrel		Eurasian Eagle–Owl		Long-Eared Owl		Barn Owl	
	Latex	µCT	Latex	µCT	Latex	µCT	Latex	µCT	Latex	µCT	Latex	µCT	Latex	µCT	Latex	µCT
Total number of feet examined for digital arteries	3	6	4	6	3	7	3	6	3	6	3	7	3	6	3	6
*Number of visible prominent digital arteries filled with latex or contrast medium*																
medial 1st toe (me_DA1)	3	6	4	6	3	7	3	6	3	6	3	7	3	6	3	6
lateral 2nd toe (la_DA2)	3	6	4	6	2	7	3	6	3	6	3	7	3	6	3	6
lateral 3rd toe (la_DA3)	3	6	4	6	3	7	3	5	3	6	3	7	3	6	3	6
medial 4th toe (me_DA4)	3	6	4	6	3	7	3	6	3	6	3	7	3	6	3	6
*Number of visible small digital arteries filled with latex or contrast medium*																
lateral 1st toe (la_DA1)	3	1	3	3	2	0	3	0	1	0	2	5	1	0	0	1
medial 2nd toe (me_DA2)	3	5	3	4	1	0	0	0	2	1	3	7	1	1	1	2
medial 3rd toe (me_DA3)	3	6	4	6	3	3	3	6	2	2	3	7	3	5	3	6
lateral 4th toe (la_DA4)	2	3	3	2	2	3	1	1	1	1	2	3	0	0	1	6

**Table 4:** Examination of digital veins: number of feet examined per avian species and investigation method (dissection after injection (latex) and contrast micro-computed tomography (µCT) scans), and number of the respective blood vessel that was visible.

	Northern Goshawk		Common Buzzard		Peregrine Falcon		Gyr × Saker Falcon		Common Kestrel		Eurasian Eagle–Owl		Long-Eared Owl		Barn Owl	
	Latex	µCT	Latex	µCT	Latex	µCT	Latex	µCT	Latex	µCT	Latex	µCT	Latex	µCT	Latex	µCT
Total number of feet examined for digital veins	3	6	6	6	3	7	4	4	1	6	3	6	4	7	4	4
*Number of visible prominent digital veins filled with latex or contrast medium*																
lateral 1st toe (la_DV1)	3	6	4	5	3	7	4	4	1	5	3	5	4	5	4	4
medial 2nd toe (me_DV2)	3	6	5	6	2	7	3	3	1	6	3	6	3	7	3	4
medial 3rd toe (me_DV3)	2	6	4	6	2	7	3	4	1	6	2	6	3	7	3	4
lateral 4th toe (la_DV4)	3	6	4	5	3	7	4	4	1	6	2	6	3	6	4	4
*Number of visible small digital veins filled with latex or contrast medium*																
medial 1st toe (me_DV1)	3	5	4	4	0	0	1	0	0	0	2	3	3	6	2	0
lateral 2nd toe (la_DV2)	2	4	2	4	1	2	3	4	1	1	2	0	3	6	3	0
lateral 3rd toe (la_DV3)	3	6	3	4	1	4	1	0	1	2	2	3	1	6	2	2
medial 4th toe (me_DV4)	2	5	3	3	1	2	2	0	1	1	2	1	2	6	2	0
*Number of visible common digital veins filled with latex or contrast medium*																
lateral dorsal (la_dCDV)	3	2	6	0	2	0	4	0	1	0	2	0	2	3	3	0
medial dorsal (me_dCDV)	3	6	4	5	3	7	3	4	1	6	2	6	3	6	3	4
medial plantar (me_pCDV)	0	0	0	0	0	0	0	0	0	0	2	1	2	6	2	0
lateral plantar (la_pCDV)	3	6	4	3	2	6	4	4	1	5	3	5	4	5	3	4

## Data Availability

Data are contained within the article.
